# Palladium-catalysed cross-coupling reaction of ultra-stabilised 2-aryl-1,3-dihydro-1*H-*benzo[*d*]1,3,2-diazaborole compounds with aryl bromides: A direct protocol for the preparation of unsymmetrical biaryls

**DOI:** 10.3762/bjoc.10.109

**Published:** 2014-05-13

**Authors:** Siphamandla Sithebe, Ross S Robinson

**Affiliations:** 1 Warren Laboratory, School of Chemistry and Physics, University of KwaZulu-Natal, Private Bag X01, Scottsville, Pietermaritzburg 3209, South Africa

**Keywords:** aryl bromide, 2-aryl-1,3-dihydro-1*H-*benzo[*d*]1,3,2-diazaborole, asymmetrical biaryls, microwave, Suzuki–Miyaura cross-coupling

## Abstract

There has been a significant interest in organoboron compounds such as arylboronic acids, arylboronate esters and potassium aryltrifluoroborate salts because they are versatile coupling partners in metal-catalysed cross-coupling reactions. On the other hand, their nitrogen analogues, namely, 1,3,2-benzodiazaborole-type compounds have been studied extensively for their intriguing absorption and fluorescence characteristics. Here we describe the first palladium-catalysed Suzuki–Miyaura cross-coupling reaction of easily accessible and ultra-stabilised 2-aryl-1,3-dihydro-1*H-*benzo[*d*]1,3,2-diazaborole derivatives with various aryl bromides. Aryl bromides bearing electron-withdrawing, electron-neutral and electron-donating substituents are reacted under the catalytic system furnishing unsymmetrical biaryl products in isolated yields of up to 96% in only 10 minutes.

## Introduction

**Figure 1:**
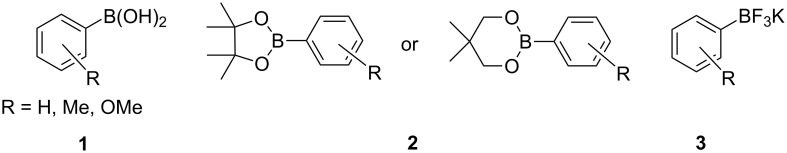
Structures of organoboron compounds **1–3**.

Arylboronic acids **1**, arylboronate esters **2** and potassium aryltrifluoroborate salts **3** (Figure 1) have received considerable attention and have found a special place as mild and versatile nucleophilic coupling partners for carbon–carbon bond-forming cross-coupling reactions [1–5]. Amongst them, the Suzuki–Miyaura cross-coupling reaction of aryl halides/triflates and organoboron compounds is one of the most documented and versatile cross-coupling reaction in the literature [6]. The use of organoboron compounds **1**, **2** and **3** (Figure 1) as nucleophilic coupling partners in the Suzuki–Miyaura cross-coupling reaction is particularly attractive due to the non-toxicity of the byproducts, the ease with which they are transmetalated and their high stability towards air and moisture, which are the key features for coupling reactions [7].

**Figure 2:**
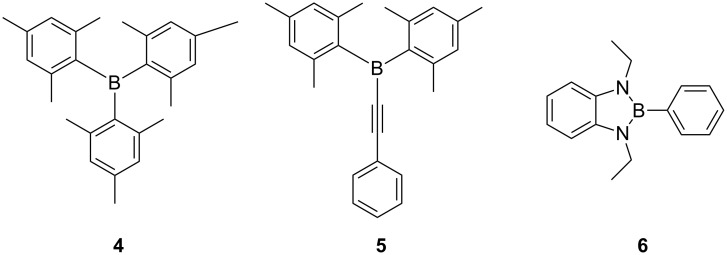
Structure of π-conjugated three-coordinate organoboron compounds **4** and **5**.

On one hand, structural diverse π-conjugated organic molecules containing a three-coordinate boron moiety such as trimesitylborane (**4**), arylalkynyldimesitylborane **5** and 2-aryl-1,3-diethyl-1*H-*benzo[*d*]1,3,2-diazaborole **6** (Figure 2) are well known and have received considerable attention due to their interesting luminescence characteristics, fluoride ion sensing abilities, emissive as well as electron-transporting properties [8–11]. Three-coordinate boron compounds are electron-poor and strong π-electron acceptors owing to the empty boron p_z_-orbital, which is capable of significant delocalisation when attached to an organic π-system [11]. These compounds exhibit an unusual stability because of the bulky aryl groups, such as mesityl (2,4,6-trimethylphenyl) groups, which provide steric conjunction around the empty boron p_z_-orbital thereby blocking the incoming nucleophile (Figure 2, compounds **4** and **5**) [12]. Alternatively, three-coordinate boron compounds functionalized with 1,3,2-benzodiazaborole units are greatly stabilised by electron back-donation from the two nitrogen atoms to the empty boron p_z_-orbital (Figure 2, compound **6**) [13–14].

**Scheme 1:**
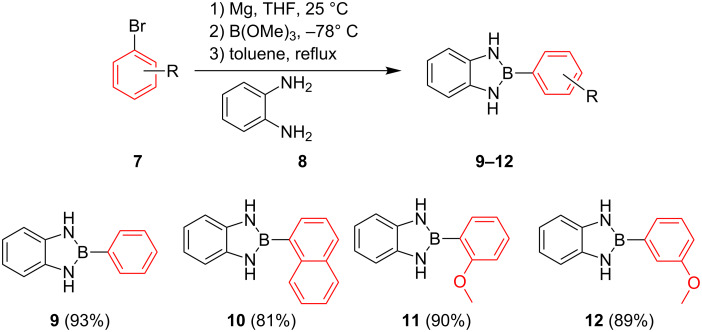
Synthesis of 2-aryl-1,3-dihydro-1*H*-benzo[*d*]-1,3,2-diazaborole compounds **9**–**12**.

Despite their popularity in the organic community, their profound stability towards air and moisture, their ease with which they are accessible and their non-toxicity, these compounds have, to the best of our knowledge, never been used in any transition metal-catalysed C–C bond formation reaction as coupling partners except for their 2-alkyl/alkenyl-substituted analogues [14]. We are aware of reports describing the Suzuki–Miyaura cross-coupling reaction aryl diaminoborole containing compounds {ArB(dan)} which are structurally similar to our compounds, however, these compounds {ArB(dan)} are used as protecting groups not as coupling partners [15–16]. During the course of our studies on the syntheses, crystal structures, fluorescence and theoretical characteristics of 1,3,2-diazaborolane functionalised organic molecules, which is reported in details elsewhere [17], we were encouraged by the high yields of 2-aryl-1,3-dihydro-1*H-*benzo[*d*]1,3,2-diazaborole compounds (Scheme 1), their solubility in various organic solvents and their high stability towards air and moisture to investigate their reactivity in transition metal-catalysed cross-coupling reaction. These compounds could be left on a bench top in a basic media for weeks without any noticeable degradation [17]. Herein, we report the first palladium-catalyzed cross-coupling reaction of 2-aryl-1,3-dihydro-1*H-*benzo[*d*]1,3,2-diazaborole compounds with aryl bromides in only 10 minutes.

## Results and Discussion

As shown in Scheme 1, 2-aryl-1,3-dihydro-1*H-*benzo[*d*]1,3,2-diazaborole compounds were easily prepared from aryl halides **7** via the reaction of an organomagnesium intermediate with trialkylborate solution followed by complexation with *o-*phenylenediamine (**8**) in a single pot (Scheme 1). Following this procedure, the desired products were obtained in excellent yields (81–93%) (Scheme 1).

### Suzuki–Miyaura cross-coupling reaction

To find optimal reaction conditions, we initially studied the reaction of bromobenzene (**13a**) with compound **9** in a toluene/water mixture under different conditions as a model reaction (Table 1). Attempted cross-coupling reaction of compound **9** with bromobenzene (**13a**), in the absence of both the ligand and a base, gave, as expected, zero conversion of the starting materials (Table 1, entry 1).

**Table 1:** Initial optimisation of reaction conditions.^a^

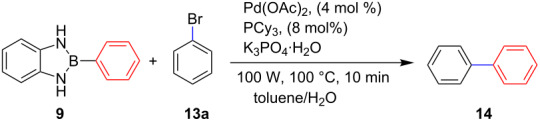				

Entry	Pd cat.	Ligand	Base	Yields (%)^b^ **13**

1	PdCl_2_	none	none	0
2	PdCl_2_	PPh_3_	K_3_PO_4_	<5
3	Pd(PPh_3_)_4_	none	K_2_CO_3_	21
4	Pd(PPh_3_)_2_Cl_2_	none	K_2_CO_3_	18
5	Pd(PPh_3_)_4_	none	K_3_PO_4_·H_2_O	10
6	Pd(PPh_3_)_2_Cl_2_	PCy_3_	K_3_PO_4_·H_2_O	33
7	PdCl_2_	PCy_3_	K_3_PO_4_·H_2_O	<5
8	Pd(OAc)_2_	PPh_3_	K_2_CO_3_	51
9	Pd(OAc)_2_	PCy_3_	K_3_PO_4_·H_2_O	88
10	Pd(OAc)_2_	PCy_3_/PPh_3_^c^	K_3_PO_4_	67
11	Pd(OAc)_2_	PCy_3_	K_2_CO_3_	76
12	Pd (PPh_3_)_2_Cl_2_	PPh_3_	K_2_CO_3_	20

^a^Reaction conditions: compound **9** (0. 85 mmol), bromobenzene (**13a**, 0.77 mmol), Pd cat. (4 mol %), ligand (8 mol %), base (3 equiv), toluene (0.50 mL) and water (0.1 mL). Closed vessel, 80 W of microwave energy, 100 °C, 100 psi of pressure, 10 minutes. ^b^Isolated yields after column chromatography. ^c^4 mol % each of the ligands.

The addition of PPh_3_ as a ligand and K_3_PO_4_ as a base failed to afford the desired coupled product in any substantive yield (Table 1, entry 2). Poor conversion of the starting material and low assay yield of the desired product were observed when more bulky Pd(PPh_3_)_4_ as a catalyst was used (Table 1, entries, 3 and 5). The use of Pd(PPh_3_)_2_Cl_2_ as a catalyst in conjunction with PPh_3_ as a ligand and K_2_CO_3_ or K_3_PO_4_·H_2_O as bases also failed to optimise the reaction conditions (Table 1, entries 4, 6 and 12). This was attributed to the decomposition of Pd(PPh_3_)_2_Cl_2_ catalyst to Pd-black. Moderate to good yields were obtained when of Pd(OAc)_2_/PCy_3_ or Pd(OAc)_2_/PPh_3_ combinations were used (Table 1, entries 8–11). Pd(OAc)_2_/PCy_3_ and K_3_PO_4_·H_2_O (Table 1, entry 9) was recognised to be the most effective combination and was thus chosen as optimal reaction conditions for the purpose of this study. With the optimized reaction conditions in hand, the scope and the limitations of the cross-coupling reaction was investigated using bromobenzene (**13a**) and 4-bromoanisole (**13b**) as elelctrophilic coupling partners and boronates **9–12** (Scheme 1) as the corresponding nucleophilic coupling partners (Table 2). The cross-coupling reaction of bromobenzene (**13a**) with boronates **9–12** went smooth affording the coupled products in yields ranging from 68% to 88% (Table 2, entries 1, 3, 5 and 7). The sterically hindered *ortho-*substituted boronate **11** generally afforded lower yields when compared to other boronate derivatives (Table 2, entries 5 and 6). This was attributed to incomplete conversion of the starting materials possibly due to a steric effect around the boron atom which is consistent with the literature [6,18–19]. The cross-coupling of an electron-rich aromatic system (4-bromoanisole) was generally less efficient compared to bromobenzene (Table 2, entries 2 and 6). This effect was attributed to the deactivation of the carbon–bromine bond as a result of electron-donating substituent (OMe) [20].

**Table 2:** Palladium-catalysed cross-coupling of boronate **9**–**12** with bromobenzene and 4-bromoanisole.^a^

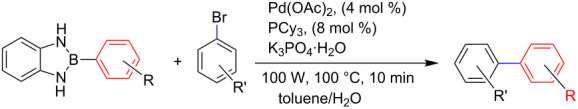				

Entry	Boronate	ArBr	Product	Yields^b^

1	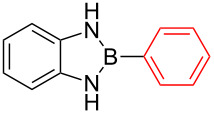 **9**	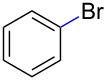 **13a**	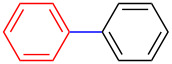 **14**	88
2	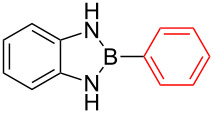 **9**	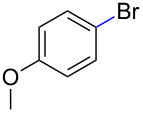 **13b**	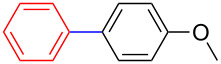 **15**	62
3	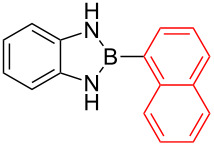 **10**	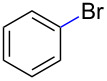 **13a**	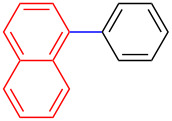 **16**	85
4	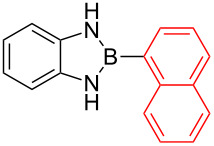 **10**	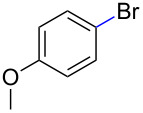 **13b**	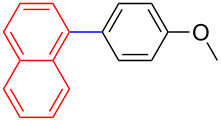 **17**	68
5	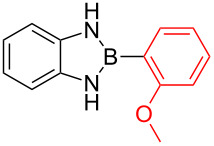 **11**	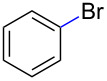 **13a**	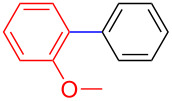 **18**	68
6	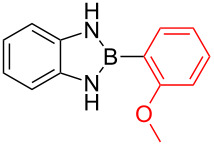 **11**	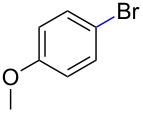 **13b**	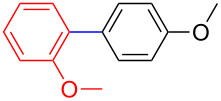 **19**	65
7	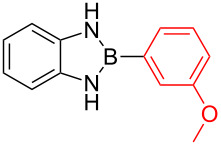 **12**	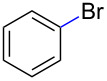 **13a**	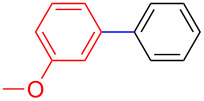 **20**	72
8	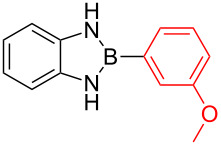 **12**	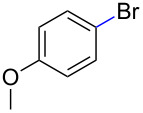 **13b**	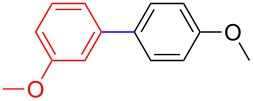 **21**	76

^a^Reaction conditions: Boronate **9–12** (1.1 eqiuv), **13a** and **13b** (1.0 equiv), Pd(OAc)_2_ (4.0 mol %), PCy_3_, (8.0 mol %), K_2_PO_4_·H_2_O (3.0 equiv), toluene (0.50 mL) and H_2_O (0.10 mL), 80 W of microwave energy, 100 °C, 100 psi, 10 minutes. ^b^Yields of isolated products after a flash column chromatography.

Encouraged by our results (Table 2), we then turned our attention to investigate the reactivity of activated as well as conjugated electrophiles in our catalytic system (Table 3). Unlike bromobenzene and 4-bromoanisole, the cross-coupling reaction of electron-deficient electrophiles (4-bromoacetophenone (**13d**) and 4-bromonitrobenzene (**13c**)) furnished the desired coupled products in excellent yields ranging from 85 to 96% (Table 3) [6,21]. These observations are consistent with the literature and are attributed to the activation of the carbon–bromine bonds due to the electron-withdrawing functional groups. The presence of an electron-withdrawing group induces oxidative addition of the carbon–bromine bond to the metal centre (catalyst) compared to the corresponding electron-neutral and electron-rich functionalities [22]. The cross-coupling reaction of boronate **9** with both substrates **13c** and **13d** afforded the desired products, as expected, in high yields (Table 3, entries 1 and 2). We noticed that steric hinderance on the boronate **10** did not have any negative impact on the cross-coupling reaction investigated herein. Sterically hindered boronate **10** was smoothly coupled with 4-bromonitrobenzene (**13c**) and 4-bromoacetophenone (**13d**) providing coupled products **24** and **25** in 96 and 94% yields, respectively (Table 3, entries 3 and 4). Although it is known that *ortho-*substituted boron counterparts usually suffer from facile protodeborination providing coupled products in low yields [5], *ortho*-substituted boronate **11** afforded the desired products **26** and **27** in 85 and 86% yields, respectively (Table 3, entries 5 and 6). The cross-coupling reaction of boronate **12**, with each of the substrates (**13c** and **13d**), furnished the desired products in 91 and 86% yields (Table 3, entries 7 and 8). The reaction was more sensitive towards increasing steric hindrance on the electrophilic counterpart (**13e**) only affording the coupled products in moderate yields (Table 3, entries 9–11).

**Table 3:** Palladium-catalysed cross-coupling of boronate **9**–**12** with 4-bromonitrobenzene, 4-bromoacetophenone and 9-bromoantracene.^a^

Entry	Boronate	ArBr	Product	Yields (%)^b^

1	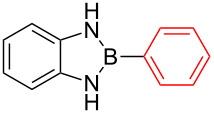 **9**	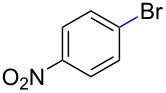 **13c**	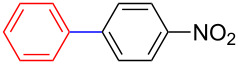 **22**	91
2	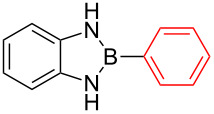 **9**	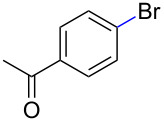 **13d**	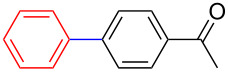 **23**	90
3	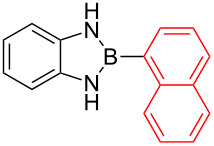 **10**	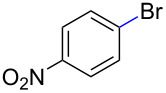 **13c**	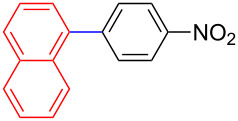 **24**	96
4	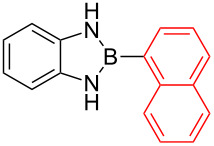 **10**	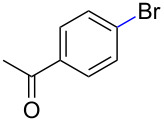 **13d**	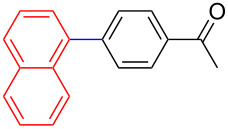 **25**	94
5	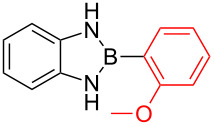 **11**	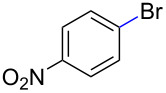 **13c**	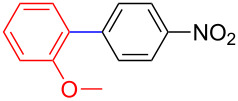 **26**	85
6	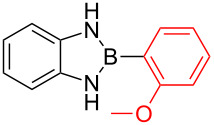 **11**	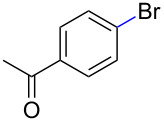 **13d**	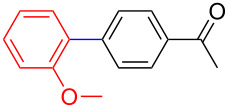 **27**	86
7	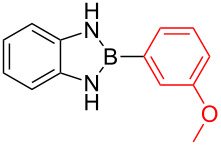 **12**	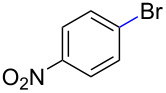 **13c**	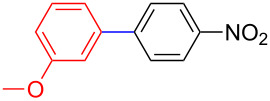 **28**	91
8	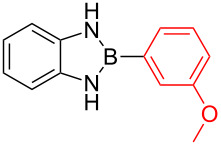 **12**	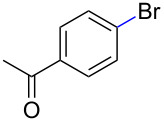 **13d**	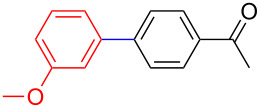 **29**	86
9	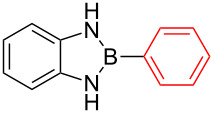 **9**	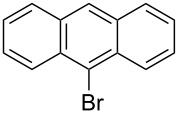 **13e**	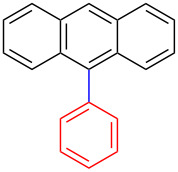 **30**	75
10	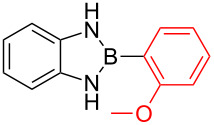 **11**	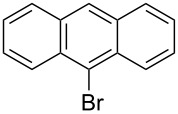 **13e**	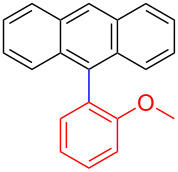 **31**	72
11	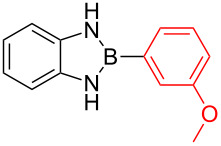 **12**	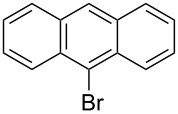 **13e**	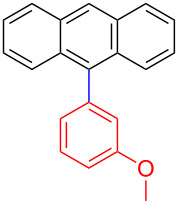 **32**	69

^a^Reaction conditions: boronate **9–12** (1.1 eqiuv), **13c–e** (1.0 equiv), Pd(OAc)_2_ (4.0 mol %), PCy_3_, (8.0 mol %), K_2_PO_4_·H_2_O (3.0 equiv), toluene (0.50 mL) and H_2_O (0.10 mL), 80 W of microwave energy, 100 °C, 100 psi, 10 minutes. ^b^Yields of isolated products after a flash column chromatography.

## Conclusion

Although arylboronic acids, arylboronate esters and potassium aryltrifluoroborate salts are powerful coupling partners in the Suzuki–Miyaura cross-coupling realm, extending the scope of organoboron compounds that can participate effectively as coupling partners in the cross-coupling reaction is still necessary. We have synthesised a range of 2-aryl-1,3-dihydro-1*H-*benzo[*d*]1,3,2-diazaborole compounds and developed their first Pd-catalysed Suzuki–Miyaura cross-coupling reaction with a range of aryl bromides bearing electron-rich, electron-neutral and electron-deficient functionalities using cost-effective and commercially available combination of Pd(OAc)_2_/PCy_3_ as a catalyst and K_3_PO_4_·H_2_O as a base. The catalytic system appeared versatile and general, tolerating a large range of functional groups such as NO_2_, OMe, COMe and diazaborolyl whilst furnishing the coupled product with isolated yields of up to 96% in only 10 minutes.

## Supporting Information

File 1 Detailed experimental procedures and copies of ^1^H and ^13^C NMR spectra of all synthesised compounds.
