# Supplementary material for: Palladium-catalysed cross-coupling reaction of ultra-stabilised 2-aryl-1,3-dihydro-1H-benzo[d]1,3,2-diazaborole compounds with aryl bromides: A direct protocol for the preparation of unsymmetrical biaryls
Source: Beilstein J Org Chem. 2014 May 13;10:1107–13. doi: 10.3762/bjoc.10.109 (PMC4077391; doi:10.3762/bjoc.10.109)
Supplement: File 1 — Detailed experimental procedures and copies of 1H and 13C NMR spectra of all synthesised compounds. [file Beilstein_J_Org_Chem-10-1107-s001.pdf]

Supporting Information

for

Palladium-catalysed cross-coupling reaction of ultra-stabilised 2-aryl-1,3-dihydro-1*H*-benzo[*d*]1,3,2-diazaborole compounds with aryl bromides: A direct protocol for the preparation of unsymmetrical biaryls

Siphamandla Sithebe and Ross S. Robinson*

Address: Warren Laboratory, School of Chemistry and Physics, University of KwaZulu-Natal, Private Bag X01, Scottsville, Pietermaritzburg 3209, South Africa

Email: Ross R. Robinson - RobinsonR@ukzn.ac.za

*Corresponding author

Detailed experimental procedures and copies of ^{1}H and ^{13}C NMR spectra of all synthesised compounds

**Content**	**Page**
**General consideration**	**S2**
• General procedure for the preparation of 2-aryl-1,3-dihydro-1*H*-benzo[*d*]1,3,2-diazaborole compounds	S3–S5
• General procedure for the Suzuki–Miyaura cross-coupling of aryl bromides with 2-aryl-1,3-dihydro-1*H*-benzo[*d*]1,3,2-diazaborole compounds	S5–S9
• NMR spectra of 2-aryl-1,3-dihydro-1*H*-benzo[*d*]1,3,2-diazaborole compounds	S9–S15
• NMR spectra of coupled products	S16–S38

General consideration:

Preparation of 2-aryl-1,3-dihydro-*1H*-benzo[*d*]1,3,2-diazaborole compounds were carried out under nitrogen atmosphere in an oven-dried glassware containing a magnetic stir bar capped with a dry rubber septa. Toluene was freshly distilled from sodium/benzophenone prior to use. Microwave reactions were performed in a CEM Discover synthetic microwave using a 10 cm microwave tube equipped with a magnetic stir bar and a vessel cap. ^{1}H NMR (400 MHz), ^{13}C NMR (100 MHz) and ^{11}B NMR (128 MHz) were recorded on Varian Bruker Avance III 400 (9.4 T) spectrometer in normal glass NMR tubes. NMR spectra were recorded as solution in the specified deuterated solvents and are reported in parts per million (ppm) downfield from tetramethylsilane (TMS) as an internal standard. ^{11}B NMR spectra were referenced to $BF_3 \cdot OEt_2$ (external, neat, with capillary tube of acetone-d_6 for the deuterium lock). Melting points were measured on a Reichert Austria apparatus using 22 × 22 mm deck Glaser.

High-resolution mass spectra (HRMS) were obtained on a Waters Acquits LCT premier (TOF) ultra performance liquid chromatography–mass spectrometry. Low resolution (Electron Impact) mass spectra were acquired on a Thermo Finnigan trace GC, coupled with a Polaris Q mass spectrometer. Infrared spectra were recorded using ID, Fourier Transform Infrared instrument. Samples were placed on a diamond and compressed with Infrared pressure steel. Purifications of the products were performed by centrifugal preparative thin-layer chromatography (chromatotron) and flash-column chromatography on Merk silica gel cat. No. 1.07749 and Fluka silica gel 60 cat No. 70–230 mesh (0.063–0.2 mm), respectively.

General procedure for the preparation of 2-aryl-1,3-dihydro-1*H*-benzo[*d*]1,3,2-diazaborole compounds

Aryl halide (8.20 mmol), Mg (398.0 mg, 16.40 mmol) and dry THF (50.0 mL) were placed in a 100 mL round-bottomed flask equipped with a Dean and Stark Apparatus, magnetic stir bar and reflux condenser. The mixture was stirred at room temperature until Grignard reagent has formed completely (minimum 30 minutes) and eventually cooled to –78 °C. Trimethyl borate solution (1.83 mL, 16.40 mmol) was added dropwise at –78 °C and the mixture was stirred overnight. THF was removed under reduced pressure leaving a white precipitate which was dissolved in toluene (50.0 mL) followed by the addition of *o*-phenylenediamine (87.0 mg, 8.20 mmol). The resulting mixture was heated to reflux for 3 h and eventually heated to dryness. The resulting brown residue was dissolved in acetone and purified by flash column and radial chromatography with hexane/ethyl acetate (8:2) mixture as an eluent.

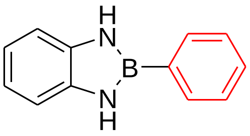

9

2-Phenyl-1,3-dihydro-1*H*-benzo[*d*]1,3,2-diazaborole (9). Following the general procedure for the preparation of 2-aryl-1,3-dihydro-*1H*-benzo[*d*]1,3,2-diazaborole, compound **9** was obtained as colorless crystals (1480.0 mg, 93%): mp 213-214 °C (lit.[1]: 212-214 °C). ^{1}H NMR (400 MHz, $DCCl_3$) δ: 6.80 (br. s, 2×*NH*), 6.83-6.89 (m, 2H), 7.09-7.14 (m, 2H), 7.39-7.44 (m, 3H), 7.90-7.95 (m, 2H). ^{13}C{^{1}H} NMR (100 MHz, $DCCl_3$) δ: 111.1, 119.4, 128.1, 129.7, 133.0, 136.3. ^{11}B NMR (128 MHz, $DCCl_3$) δ: 28.8

[1] Soloway, A. H. *J. Am. Chem. Soc.***1959**, *82*, 2442-2444.

{s, C*B*(NH)$_2$}. IR (neat): 3441, 3418, 3056 cm^{-1}. HRMS: found [M$^+$] 193.0938, calculated for $C_{12}H_{10}BN_2$ 193.0937.

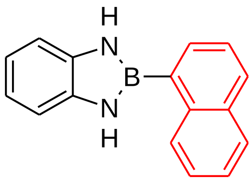

10

2-(1-Naphthyl)-1,3-dihydro-1*H*-benzo[*d*]1,3,2-diazaborole (10). Following the general procedure for the preparation of 2-aryl-1,3-dihydro-1*H*-benzo[*d*]1,3,2-diazaboroles, **10** was obtained as white crystals (1620.0 mg, 81%): mp 149-152 °C (lit.[2]: 149-150 °C). ^{1}H NMR (400 MHz,CDCl$_3$) δ: 6.88 (br. s, 2×*NH*), 7.02-7.08 (m, 2H), 7.18-7.23 (m, 2H), 7.51-7.56 (m, 3H), 7.81 (dd, *J* = 6.8, 1.2 Hz, 1H), 7.90-7.97 (m, 2H), 8.26-8.32 (m, 1H). ^{13}C{^{1}H} NMR (100 MHz,CDCl$_3$) δ: 111.2, 119.5, 125.3, 125.7, 126.2, 128.1, 128.7, 129.5, 132.8, 133.3, 136.1. ^{11}B NMR (128 MHz,CDCl$_3$), δ: 29.4 {s, C*B*(NH)$_2$}. IR (neat): 3443, 3422, 3048 cm^{-1}. HRMS: found 243.1099 [M$^+$], calculated for $C_{16}H_{12}BN_2$ 243.1094.

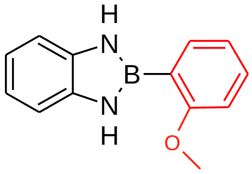

11

2-(2-Methoxyphenyl)-1,3-dihydro-1*H*-benzo[*d*]1,3,2-diazaborole (11). Following the general procedure for the preparation of 2-aryl-1,3-dihydro-1*H*-benzo[*d*]1,3,2-diazaboroles, **11** was obtained as as cream white crystals (1540.0 mg, 84%): mp 118-120 °C. ^{1}H NMR (400 MHz,CDCl$_3$) δ: 3.98 (s, 3H), 6.95-7.01 (m, 3H), 7.04-7.10 (m, 3H), 7.12-7.18 (m, 2H), 7.40-7.47 (m, 1H), 7.70 (dd, *J* = 7.3, 1.4 Hz, 1H). ^{13}C{^{1}H} NMR (100 MHz,CDCl$_3$) δ: 55.3, 110.3, 110.9, 119.0, 120.6, 131.2, 134.8, 136.2, 163.6. ^{11}B NMR

(128 MHz, $CDCl_3$) δ: 27.6 {s, $CB(NH)_2$}. IR (neat): 3467, 3423, 1598 cm^{-1}. HRMS: found [M^+] 223.1045, calculated for $C_{13}H_{12}BN_2O$ 223.1043.

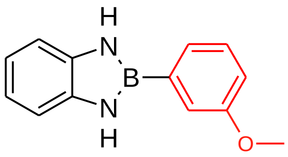

12

2-(3-Methoxyphenyl)-1,3-dihydro-1*H*-benzo[*d*]1,3,2-diazaborole (12). Following the general procedure for the preparation of 2-aryl-1,3-dihydro-1*H*-benzo[*d*]1,3,2-diazaboroles, **12** was obtained as cream white crystals (1370 mg, 89%): mp 132-133 °C. ^{1}H NMR (400 MHz, Acetone-d_6) δ: 3.85 (s, 3H), 6.83-6.89 (m, 2H), 6.98 (dd, J = 8.1, 2.5 Hz, 1H), 7.08-7.14 (m, 2H), 7.34 (t, J = 7.8 Hz, 1H), 7.47-7.55 (m, 2H), 8.44 (br. s, 2×*NH*). $^{13}C\{^{1}H\}$ NMR (100 MHz, Acetone-d_6) δ: 54.5, 107.7, 110.9, 115.0, 118.6, 118.6, 125.5, 129.0, 137.2, 159.5. ^{11}B NMR (128 MHz, Acetone-d_6) δ: 23.5 {s, $CB(NH)_2$}. IR (neat): 3414, 3052, 29.23 cm^{-1}. HRMS: found [M^+] 223.1043, calculated for $C_{13}H_{12}BN_2O$ 223.1043.

General procedure for the Suzuki–Miyaura cross-coupling reaction.

A microwave tube equipped with a magnetic stir bar was charged with the corresponding 2-aryl-1,3-dihydro-1*H*-benzo[*d*]1,3,2-diazaborole (0.85 mmol, 1.1 equiv) $Pd(OAc)_2$ (6.91 mg, 0.031 mmol, 4.0 mol %), PCy_3 (17.39 mg, 0.062 mmol, 8.0 mol %), aryl halide (0.77 mmol, 1.0 equiv) $K_3PO_4{\cdot}H_2O$ (530.0 mg, 2.31 mmol, 3.0 equiv), toluene (0.5 0 mL) and water (0.1 mL). The flask was fitted with a rubber septum and argon was bubbled through the solution for 20 minutes. The microwave tube was irradiated, in a closed vessel, with 80 W of microwave energy at 100 psi and 100 °C for 10 minutes. The

reaction mixture was diluted with dicloromethane (5.0 mL), filtered and concentrated under reduced pressure. The resulting black residue was purified through a flash and radial chromatography using hexane/ethyl acetate (9:1) as an eluting solvent.

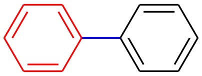

14

Biphenyl (14): Following the general procedure, 2-phenyl-1,3-dihydro-1*H*-benzo[*d*]1,3,2-diazaborole (**9**, 164.9 mg, 0.85 mmol) was coupled with bromobenzene (**13a**, 120.9 mg, 0.77 mmol) to afford **14** as cream white crystalline product (104.5 mg, 88%): mp 69-70 °C (lit. [2]: 69-70 °C). ^{1}H NMR (400 MHz, $CDCl_3$) δ: 7.34-7.40 (m, 2H), 7.42-7.50 (m, 4H), 7.59-7.64 (m, 4H). ^{13}C{^{1}H} NMR (100 MHz, $CDCl_3$) δ: 127.1, 127.2, 128.7, 141.2. MS (EIMS): *m/z* (%) 154 [M$^+$] (100%), 153 (41%), 152 (34%), 155 (14%), 151(11%).

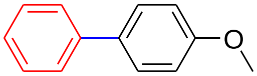

15

4-Methoxybiphenyl (15): Following the general procedure, 2-phenyl-1,3-dihydro-1*H*-benzo[*d*]1,3,2-diazaborole (**9**, 164.9 mg, 0.85 mmol) was reacted with 4-bromoanisole (**13b**, 144.0 mg, 0.77 mmol) to afford **15** as a white powder (87.9 mg, 62%): mp 90-91°C (lit. [3]: 90-91°C). ^{1}H NMR (400 MHz, $CDCl_3$) δ: 3.89 (s, 3H), 7.00 (d, *J* = 8.9 Hz, 2H),7.31-7.39 (m, 1H) 7.43 (t, *J* = 7.8 Hz, 2H), 7.55-7.64 (m, 4H).^{13}C {^{1}H} NMR (100 MHz, $CDCl_3$) δ: 55.3, 114.2, 126.6, 126.7, 128.1, 128.7, 133.8, 140.8, 159.2. MS

[2] Zhang, L.; Wang, L.; Li, H.; Li, P. *Synth. Commun.* **2008**, *38*, 1498-1511.
[3] Desmarets, C.; Omar-Amrani, R.; Walcarius, A.; Lambert, J.; Champagne, B.; Fort, Y.; Schneider, R. *Tetrahedron* **2008**, *64*, 372-381.

(EIMS) *m/z* (%): 184 [M^+] (100%), 169 (77%), 141 (60%), 115 (32%), 185 [M^+ + 1] (15%).

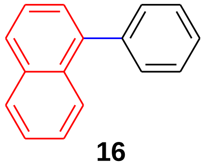

16

1-Phenylnaphthalene (16): Following the general procedure, 2-(1-naphthyl)-1,3-dihydro-1*H*-benzo[*d*]1,3,2-diazaborole (**10**, 207.5 mg, 0.85 mmol) was reacted with bromobenzene (**13a**, 120.9 mg, 0.77 mmol) to afford **16** as a colorless oil[4] (133.7 mg, 85%). ^{1}H NMR (400 MHz, $CDCl_3$) δ: 7.43-7.48 (m, 3H), 7.48-7.57 (m, 6H), 7.89 (d, *J* = 8.2 Hz, 1H), 7.90-7.96 (m, 2H). ^{13}C{^{1}H} NMR (100 MHz, $CDCl_3$) δ:125.3, 125.7, 126.0, 126.0, 126.9, 127.2, 127.6, 128.2, 130.0, 131.6, 133.8, 140.2, 140.7. MS (EIMS) *m/z* (%):101 (6), 200 (9), 202 (45), 204[M^+] (100), 205 [M^++1].

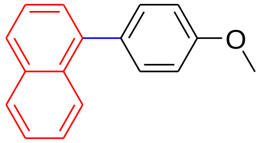

17

1-(4-Methoxyphenyl)naphthalene (17): Following the general procedure, 2-(1-naphthyl)-1,3-dihydro-1*H*-benzo[*d*]1,3,2-diazaborole (**10**, 207.5 mg, 0.85 mmol) was reacted with 4-bromoanisole (**13b**, 144.0 mg, 0.77 mmol) to afford **17** as colorless crystals (122.7 mg, 68%): mp 116-117 °C (lit.[5]: 116-117 °C). ^{1}H NMR (400 MHz, $CDCl_3$) δ : 3.92 (s, 3H), 7.05 (d, *J* = 8.7 Hz, 2H), 7.40-7.47 (m, 4H), 7.47-7.55 (m, 2H), 7.85 (d, *J* = 8.3 Hz, 1H), 7.93 (t, *J* = 9.1 Hz, 2H). ^{13}C{^{1}H} NMR (100 MHz, $CDCl_3$) δ :

---

[4] Nandurkar, N. S.; Bhanage, B. M. *Tetrahedron* **2008**, *64,* 3655-3660.
[5] Wang, L.; Lu, W. *Org. Lett.* **2009**, *11*, 1079-1082.

55.3, 113.8, 125.4, 125.7, 125.9, 126.1, 126.9, 127.3, 128.3, 131.1, 133.2, 133.9, 139.9, 140.0, 159.0.

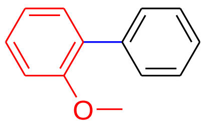

18

2-Methoxybiphenyl (18): Following the general procedure, 2-(2-methoxyphenyl)-1,3-dihydro-1*H*-benzo[*d*]1,3,2-diazaborole (**11**, 190.5 mg, 0.85 mmol) was reacted with bromobenzene (**13a**, 120.9 mg, 0.77 mmol) to afford **18** as a light yellow oil[9,10] (96.5 mg, 68%): ^{1}H NMR (400 MHz, $CDCl_3$) δ: 3.85 (s, 3H), 7.01-7.10 (m, 2H), 7. 33-7.39 (m, 3H), 7.42-7.48 (m, 2H), 7.55-7.60 (m, 2H). ^{13}C{^{1}H} NMR (100 MHz, $CDCl_3$) δ: 55.5, 111.3, 120.8, 126.9, 127.9, 128.6, 129.5, 130.8, 130.9, 138.5, 156.5. MS (EIMS) *m/z* (%): 184 [M^+] (100%), 141 (57%), 169 (53%), 115 (39%), 168 (14%), 185 [M^+ + 1] (13%).

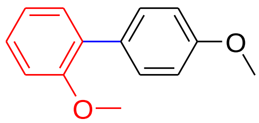

19

1,1'-Biphenyl,2,4'dimethoxy-(19): Following the general procedure, 2-(2-methoxyphenyl)-1,3-dihydro-1*H*-benzo[*d*]1,3,2-diazaborole (**11**, 190.5 mg, 0.85 mmol) was reacted with 4-bromoanisole (**13b**, 144.0 mg, 0.77 mmol) to afford **19** as a white powder (107.2 mg, 65%) mp 69-70 °C (lit.[6]: 70-71°C) : ^{1}H NMR (400 MHz, $CDCl_3$) δ : 3.83 (3H, s), 3.87 (s, 3H), 6.94-7.04 (m, 4H), 7.28-7.36 (m, 2H), 7.50 (d, J = 8.8 Hz, 2H). ^{13}C{^{1}H} NMR (100 MHz, $CDCl_3$) δ : 55.2, 55.5, 111.3, 113.5, 120.8, 128.1, 130.4, 130.6, 130.7, 130.9, 156.5, 158.7. MS (EIMS) *m/z* (%): 214 [M^+] (100%), 199 (56%), 184 (44%), 128 (23%), 215 [M^+ + 1] (15%).

[6] Qin, C.; Lu, W. *J. Org. Chem.* **2008**, *73*, 7424-7427.

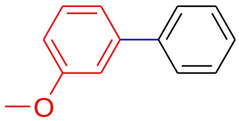

20

3-Methoxybiphenyl (20): Following the general procedure, 2-(3-methoxyphenyl)-1,3-dihydro-1*H*-benzo[*d*]1,3,2-diazaborole (**12**, 190.5 mg, 0.85 mmol) was reacted with bromobenzene (**13b**, 120.9 mg, 0.77 mmol) to afford **20** as a colorless oil[7,8] (102.1 mg, 72%): ^{1}H NMR (400 MHz, $CDCl_3$) δ: 3.90 (s, 3H), 6.93 (dd, *J* = 8.3, 2.6 Hz, 1H), 7.15-7.18 (m, 1H), 7.20-7.24 (m, 1H), 7.36-7.42 (m, 2H), 7.44-7.50 (m, 2H), 7.60-7.65 (m, 2H). ^{13}C{^{1}H} NMR (100 MHz, $CDCl_3$) δ : 55.3, 112.7, 112.9, 119.7, 127.2, 127.4, 128.7, 129.7, 141.1, 142.8, 159.9. MS (EIMS) *m/z* (%): 184 [M^+] (100%), 154 (38%), 115 (24%), 155 (24%), 115 (23%), 153 (21%), 141 (16%), 185 [M^+ + 1] (15%).

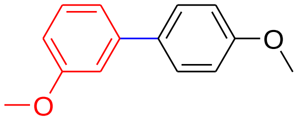

21

3,4'-Dimethoxybiphenyl (21): Following the general procedure, 2-(3-methoxyphenyl)-1,3-dihydro-1*H*-benzo[*d*]1,3,2-diazaborole (**12**, 190.5 mg, 0.85 mmol) was reacted with 4-bromoanisole (**13b**, 144.0 mg, 0.77 mmol) to afford **21** as a white powder (125.3 mg, 76%): mp 58-59 °C (lit.[9]: 58-60 °C): ^{1}H NMR (400 MHz, $CDCl_3$) δ : 3.88 (s, 3H), 3.89 (s, 3H), 6.88 (dd, *J* = 8.1, 1.7 Hz, 1H), 7.00 (d, *J* = 8.7 Hz, 2H), 7.10-7.13 (m, 1H), 7.17 (d, *J* = 7.8 Hz, 1H), 7.36 (t, *J* = 8.0 Hz, 1H), 7.55 (d, *J* = 8.7 Hz, 2H).^{13}C{^{1}H} NMR (100 MHz, $CDCl_3$) δ : 55.2, 55.3, 112.0, 112.5, 114.2, 119.3, 128.1, 129.7, 133.6, 142.3, 159.2, 159.9. MS (EIMS) *m/z* (%): 214 [M^+] (100%), 199 (56%), 184 (44%), 128 (23%), 215 [M^+ + 1] (15%).

---

[7] Bolliger, J. L.; Fresh, C. M. *Chem-Eur. J.* **2010**, *16*, 4075-4081.
[8]Wei, C.; Pinhua, L.; Lui, W. *Tetrahedron* **2011**, *67,* 318-325.
[9] Itoh, Y.; Brossi, A.; Hamel, E.; Lin, C. M. *Helvetica Chimica Acta* **1988**, *71*. 1199-1209.

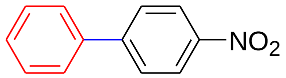

22

4-Nitrobiphenyl (22): Following the general procedure, 2-phenyl-1,3-dihydro-1*H*-benzo[*d*]1,3,2-diazaborole (**9**, 190.5 mg, 0.85 mmol) was reacted with 4-bromonitrobenzene (**13c**, 155.5 mg, 0.77 mmol) to afford **22** as colorless crystals (139.6 mg, 91%) mp 112-114 °C (lit.[10]: 112-115° C): ^{1}H NMR (400 MHz, $CDCl_3$) δ: 7.43-7.56 (m, 3H),7.61-7.67 (m, 2H), 7.76 (d, *J* = 8.9 Hz, 2H), 8.32 (d, *J* = 9.0 Hz, 2H). ^{13}C{^{1}H} NMR (100 MHz, $CDCl_3$) δ: 124.0, 127.3, 127.7, 128.9, 129.1, 138.7, 147.1, 147.6. MS (EIMS): *m/z* (%): 115 (11), 141 (41), 152 (93), 169 (70), 199 [M^+] (100), 200 [M^++1] (15).

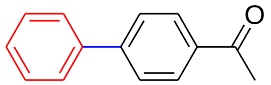

23

4-Acetylbiphenyl (23): Following the general procedure, 2-phenyl-1,3-dihydro-1*H*-benzo[*d*]1,3,2-diazaborole (**9**, 190.5 mg, 0.85 mmol) was reacted with 4-bromoacetophenone (**13d**, 153.2 mg, 0.77 mmol) to afford **23** as colorless needle-like crystals (111.8 mg, 90%): mp 123-124 °C (lit.[11]: 122-124 °C): ^{1}H NMR (400 MHz, $CDCl_3$) δ : 2.66 (s, 3H), 7.38-7.44 (m,1H), 7.44-7.52 (m, 2H), 7.65 (d, *J* = 8.2 Hz, 2H), 7.70 (d, *J* = 7.6 Hz, 2H), 8.05 (d, *J* = 7.7 Hz, 2H).^{13}C{^{1}H} NMR (100 MHz, $CDCl_3$) δ: 26.6, 127.2, 127.2, 128.2, 128.9, 128.9, 135.9, 139.9, 145.8, 197.7. MS (EIMS) *m/z* (%):181 (100%), 152 (54%), 153 (47%), 196 [M^+] (41%), 151 (16%), 197 [M^+ + 1] (9%), 154 (6%). IR (neat): 1677, 1600, cm^{-1}.

[10] Milanesi, S.; Fagnoni, M.; Albini, A. *J. Org. Chem.* **2005**, *70*, 603-610.
[11] Metallinos, C.; Barrett, F. B.; Chaytor, J. L.; Heska, M. E. A. *Org. Lett.* **2004**, *6*, 3641-3644.

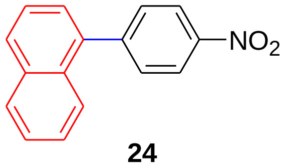

24

1-(4-Nitrophenyl)naphthalene (24): Following the general procedure, 2-(1-naphthyl)-1,3-dihydro-1*H*-benzo[*d*]1,3,2-diazaborole (**10**, 207.5 mg, 0.85 mmol) was reacted with 4-bromonitrobenzene (**13c**, 155.5 mg, 0.77 mmol) to afford **24** as colorless crystals (184.3 mg, 96%): mp 132-133 °C (lit.[7]:132-133 °C). ^{1}H NMR (400 MHz, $CDCl_3$) δ : 7.40-7.52 (m, 4H), 7.52-7.62 (m, 2H), 7.69 (d, *J* = 8.8 Hz, 2H), 7.80 (d, *J* = 8.4 Hz, 1H), 7.96 (dd, *J* = 7.8, 2.9 Hz, 2H), 8.38 (d, *J* = 8.8 Hz, 2H). ^{13}C{^{1}H} NMR (100 MHz, $CDCl_3$) δ: 123.5, 125.1, 125.3, 126.2, 126.7, 127.0, 128.5, 128.9, 130.9, 130.9, 133.8, 137.8, 147.2, 147.69. MS (EIMS) *m/z* (%): 202 (100%), 249 [M^+] (95%), 203 (59%), 203 (41%), 200 (14%), 201 (19%), 219 (16%), 250 [M^+ + 1] (13%).

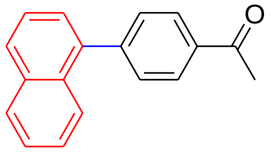

25

1-{4-(1-Naphthalenyl)phenyl}ethanone (25): Following the general procedure, 2-(1-naphthyl)-1,3-dihydro-1*H*-benzo[*d*]1,3,2-diazaborole (**10**, 207.5 mg, 0.85 mmol) was reacted with 4-bromoacetophenone (**13d**, 153.2 mg, 0.77 mmol) to afford **25** as colorless crystals (178.3 mg, 94%) mp 102-103 °C (lit.[7]: 102-103 °C): ^{1}H NMR (400 MHz, $CDCl_3$) δ: 2.70 (s, 3H), 7.42-7.50 (m, 2H), 7.50-7.60 (m, 2H), 7.63 (d, *J* = 8.3 Hz, 2H), 7.86 (d, *J* = 8.4 Hz, 1H), 7.93 (t, *J* = 9.5 Hz, 2H), 8.11 (d, *J* = 8.5 Hz, 2H).^{13}C{^{1}H} NMR (100 MHz, $CDCl_3$) δ: 26.6, 125.3, 125.5, 125.9, 126.3, 126.9, 128.3, 128.4, 130.3, 131.2, 133.8, 136.0, 139.0, 145.8. MS (EIMS) *m/z* (%): 231 (100%), 246 [M^+] (72%), 202 (64%), 203 (41%), 247 [M^++ 1] (14%), 201 (12%). IR (neat): 1681, 1604 cm^{-1}.

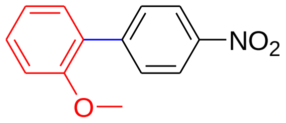

26

2-(4-Nitrophenyl)anisole (26): Following the general procedure, 2-(2-methoxyphenyl)-1,3-dihydro-1*H*-benzo[*d*]1,3,2-diazaborole (**11**, 190.5 mg, 0.85 mmol) was reacted with 4-bromonitrobezene (**13c**, 155.5 mg, 0.77 mmol) to afford **26** as a white powder (150.0 mg, 85%): mp 62-63 °C (lit.[3]:61-63 °C). ^{1}H NMR (400 MHz, $CDCl_3$) δ : 3.86 (s, 3H), 7.02-7.11 (m, 2H), 7.35 (dd, *J* = 7.5, 1.7 Hz, 1H), 7.38-7.45 (m, 1H), 7.71 (d, *J* = 8.8 Hz, 2H), 8.20 (d, *J* = 8.8 Hz, 2H). ^{13}C{^{1}H} NMR (100 MHz, $CDCl_3$) δ: 55.5, 111.4, 121.1, 123.2, 128.3, 130.1, 130.3, 130.6, 145.4, 146.6, 156.4. MS (EIMS) *m/z* (%): 229 [M^+] (100%), 139 (53%), 152 (28%), 128 (21%), 230 [M^+ + 1] (10%).

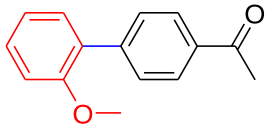

27

1-[2'-Methoxy-(1,1'-biphenyl)-4-yl)ethanone (27): Following the general procedure, 2-(2-methoxyphenyl)-1,3-dihydro-1*H*-benzo[*d*]1,3,2-diazaborole (**11**, 190.5 mg, 0.85 mmol) was reacted with 4-bromoacetophenone (**13d**, 153.2 mg, 0.77 mmol) to afford **27** as a white powder (149.8 mg, 86%): mp 105-107 °C (lit.[12]: 106-107 °C). ^{1}H NMR (400 MHz, $CDCl_3$) δ: 2.66 (s, 3H), 3.84 (s, 3H), 7.02-7.09 (m, 2H), 7.33-7.41 (m, 2H), 7.65 (d, *J* = 8.6 Hz, 2H), 8.02 (d, *J* = 8.4 Hz, 2H). ^{13}C{^{1}H} NMR (100 MHz, $CDCl_3$) δ: 26.5, 55.5, 111.4, 120.9, 128.0, 129.4, 129.7, 130.7, 135.5, 143.6, 156.5, 197.8. MS (EIMS) *m/z* (%): 211 (100%), 168 (63%), 226 [M^+] (54%), 139 (19%), 227 [M^+ + 1] (11%), 160 (9%), 152 (6%). IR (neat): 1670, 1601cm^{-1}.

[12] Quasdorf, W. K.; Riecher, M.; Petrova, V. K. Garg, K. N. *J. Am. Chem. Soc.*$\,$**2009**, *131*, 17748-17749.

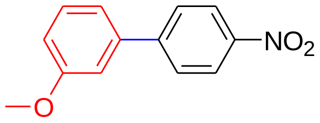

28

3-(4-Nitrophenyl)anisole (28): Following the general procedure, 2-(3-methoxyphenyl)-1,3-dihydro-1*H*-benzo[*d*]1,3,2-diazaborole (**12**, 190.5 mg, 0.85 mmol) was reacted with 4-bromonitrobenzene (**13c**, 155.5 mg, 0.77 mmol) to afford **28** as white crystals (160.6 mg, 91%): mp 86-87 °C (lit.[13]: 86-87 °C). ^{1}H NMR (400 MHz, $CDCl_3$) δ: 3.90 (s, 3H), 7.00 (dd, *J* = 8.1, 2.3 Hz, 1H), 7.13-7.7.17 (m, 1H), 7.19-7.24 (m, 1H), 7.43 (t, *J* = 8.0 Hz, 1H), 7.74 (d, *J* = 8.8 Hz, 2H), 8.31 (d, *J* = 8.9 Hz, 2H). ^{13}C{^{1}H} NMR (100 MHz, $CDCl_3$) δ: 55.4, 113.2, 114.1, 119.8, 124.0, 127.8, 130.2, 140.2, 147.2, 147.4, 160.1. MS (EIMS) *m/z* (%): 229 [M^+] (100%), 139 (53%), 171 (49%), 199 (48%), 140 (42%), 152 (28%), 128 (21%), 230 [M^+ + 1] (10%).

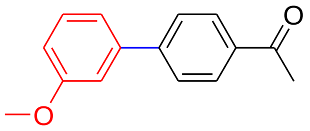

29

1-(3'-Methoxybiphenyl-4-yl)ethanone (29): Following the general procedure, 2-(3-methoxyphenyl)-1,3-dihydro-1*H*-benzo[*d*]1,3,2-diazaborole (**12**, 190.5 mg, 0.85 mmol) was reacted with 4-bromoacetophenone (**13d**, 153.2 mg, 0.77 mmol) to afford **29** as a cream white powder (190.9 mg, 86%): mp 61-63 °C (lit.[3]: 61-63 °C). ^{1}H NMR (400 MHz, $CDCl_3$) δ : 2.66 (s, 3H), 3.90 (s, 3H), 6.96 (dd, *J* = 8.4, 2.1 Hz, 1H), 7.17 (s, 1H), 7.23 (d, *J* = 7.6 Hz, 1H), 7.40 (t, *J* = 7.9 Hz, 1H), 7.69 (d, *J* = 8.3 Hz, 2H), 8.04 (d, *J* = 8.2 Hz, 2H). ^{13}C {^{1}H} NMR (100 MHz, $CDCl_3$) δ: 26.6, 55.3, 113.1, 113.5, 119.7, 127.2, 128.8, 129.9, 136.0, 141.4, 145.6, 160.0, 197.6. MS (EIMS) *m/z* (%): 211 (100%), 226

[13] Seganish, W. M.; DeShong, P. *J. Org. Chem.* **2004**, *69*, 6790-6795.

[M^+] (53%), 168 (19%), 153(19%), 139 (15%), 227 [M^+ + 1] (8%). IR (neat): 16731485, cm^{-1}.

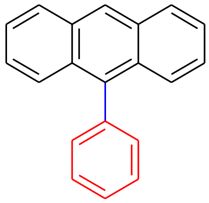

30

9-Phenylanthracene (30): Following the general procedure, 2-phenyl-1,3-dihydro-1*H*-benzo[*d*]1,3,2-diazaborole (**9**, 190.5 mg, 0.85 mmol) was reacted with 9-bromoanthracene (**13e**, 198.0 mg, 0.77 mmol) to afford **30** as colorless plates-like crystals (146.9 mg,75%): mp 155-157 °C (lit.[14]: 153-155 °C). ^{1}H NMR (400 MHz, $CDCl_3$) δ: 7.32-7.42 (m, 2H), 7.42-7.53 (m, 4H), 7.53-7.65 (m, 3H), 7.70 (d, *J* = 8.79 Hz, 2H), 8.07 (d, *J* = 8.45 Hz, 2H), 8.52 (s, 1H). ^{13}C{^{1}H} NMR (100 MHz, $CDCl_3$) δ: 125.1, 125.3, 126.5, 126.8, 127.4, 128.3, 128.3, 130.24, 131.2, 131.3, 137.0, 138.8.

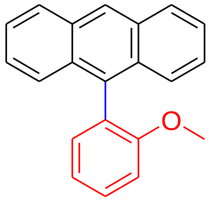

31

9-(2-Methoxyphenyl)anthracene (31): Following the general procedure, 2-(2-methoxyphenyl)-1,3-dihydro-1*H*-benzo[*d*]1,3,2-diazaborole (**11**, 190.5 mg, 0.85 mmol) was reacted with 9-bromoanthracene (**13e**, 198.0 mg, 0.77 mmol) to afford **31** as cream-white powder (157.6 mg, 72%): mp 177-178 °C (lit.[15]:177-179 °C).^{1}H NMR (400 MHz, $CDCl_3$) δ: 3.67 (s, 3H), 7.19-7.30 (m, 2H), 7.36-7.48 (m, 3H), 7.53 (t, *J* = 7.0 Hz,

---

[14] Nakamichi, N.; Kawabota, H.; Hayashi, M. *J. Org. Chem.* **2003**, *68*, 8272-8273.
[15] Yoshiba, T.; Hiroyuku, W.; Michiyo, N.; Tetsuya, S.; Masahiro, M.; Masakatsu, N. *J. Org. Chem.* **2003**, *68*, 5236-5243.

2H), 7.58-7.65 (m, 1H), 7.75 (d, J = 8.7 Hz, 2H), 8.12 (d, J = 8.3 Hz, 2H), 8.55 (s, 1H). $^{13}C\ \{^1H\}$ NMR (100 MHz, $CDCl_3$) δ: 55.7, 111.3, 120.7, 125.0, 125.2, 126.5, 126.7, 127.3, 128.4, 129.3, 130.4, 131.4, 132.8, 133.7, 158.0. MS (EIMS) *m/z* (%): 119 (10), 126 (5), 178 (20), 191 (10), 239 (36), 284 [M^+] (100), 285 [M^++1] (16).

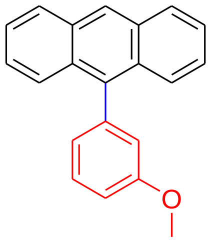

32

9-(3-Methoxyphenyl)anthracene (32): Following the general procedure, 2-(3-methoxyphenyl)-1,3-dihydro-1*H*-benzo[*d*]1,3,2-diazaborole (**12**, 190.5 mg, 0.85 mmol) was coupled with 9-bromoanthracene (**13e**, 198.0 mg, 0.77 mmol) to afford **32** as colorless crystals (151.0 mg, 69%): mp 99-101 °C (lit.[16]: 99-100 °C). ^1H NMR (400 MHz, $CDCl_3$) δ: 3.92 (s, 3H), 7.13-7.21 (m, 3H), 7.33-7.41 (m, 2H), 7.44-7.55 (m, 3H), 7.73 (d, J = 8.82 Hz, 2H), 8.06 (d, J = 8.45 Hz, 2H), 8.51 (s, 1H). $^{13}C\{^1H\}$ NMR (100 MHz, $CDCl_3$) δ: 55.3, 113.2, 116.60, 123.7, 125.1, 125.3, 126.5, 126.8, 128.2, 129.3, 130.1, 131.3, 136.8, 140.2, 159.6.

[16] Dikerman, C. S.; Klein, M. V.; George, B. *J. Org. Chem*. **1964**, *29*, 3695-3697.

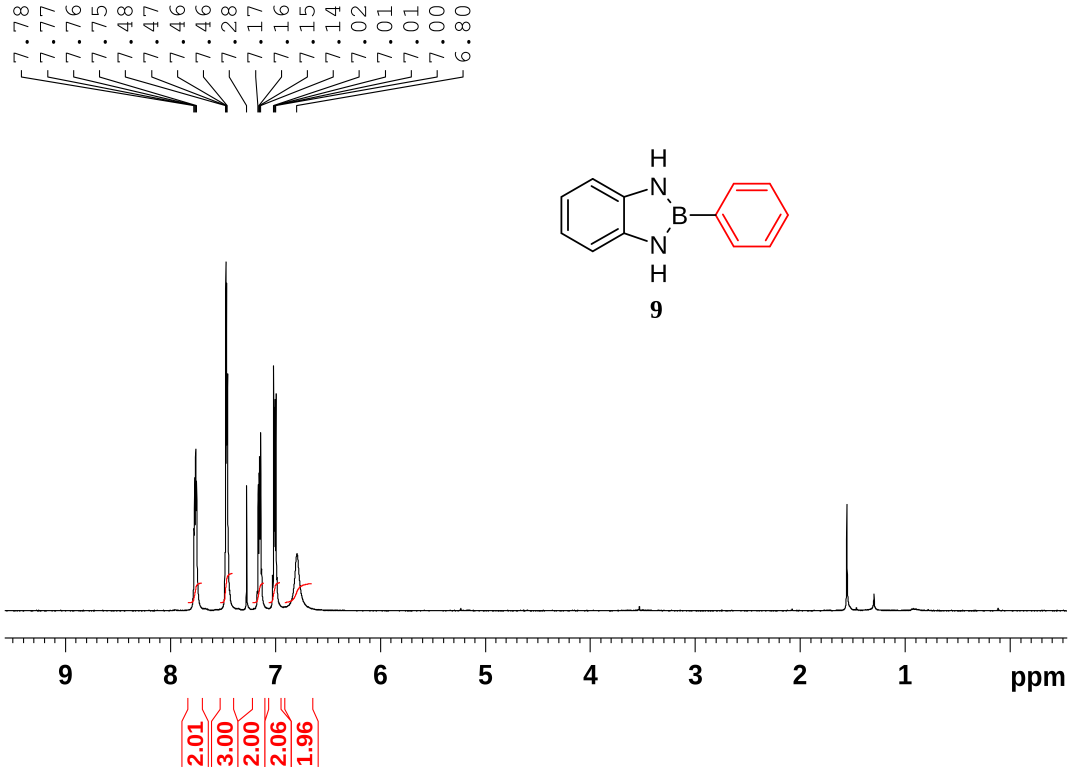
7.78
7.77
7.76
7.75
7.48
7.47
7.46
7.46
7.28
7.17
7.16
7.15
7.14
7.02
7.01
7.01
7.00
6.80
9
ppm
2.01
3.00
2.00
2.06
1.96

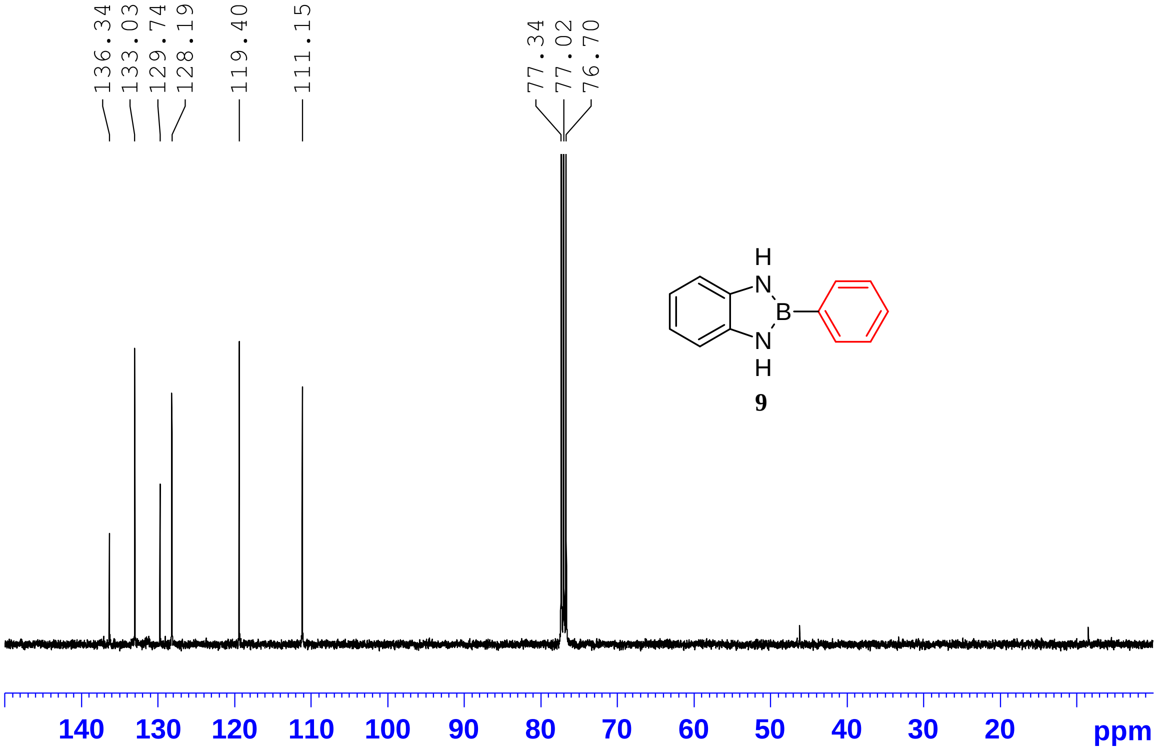
136.34
133.03
129.74
128.19
119.40
111.15
77.34
77.02
76.70
9
ppm

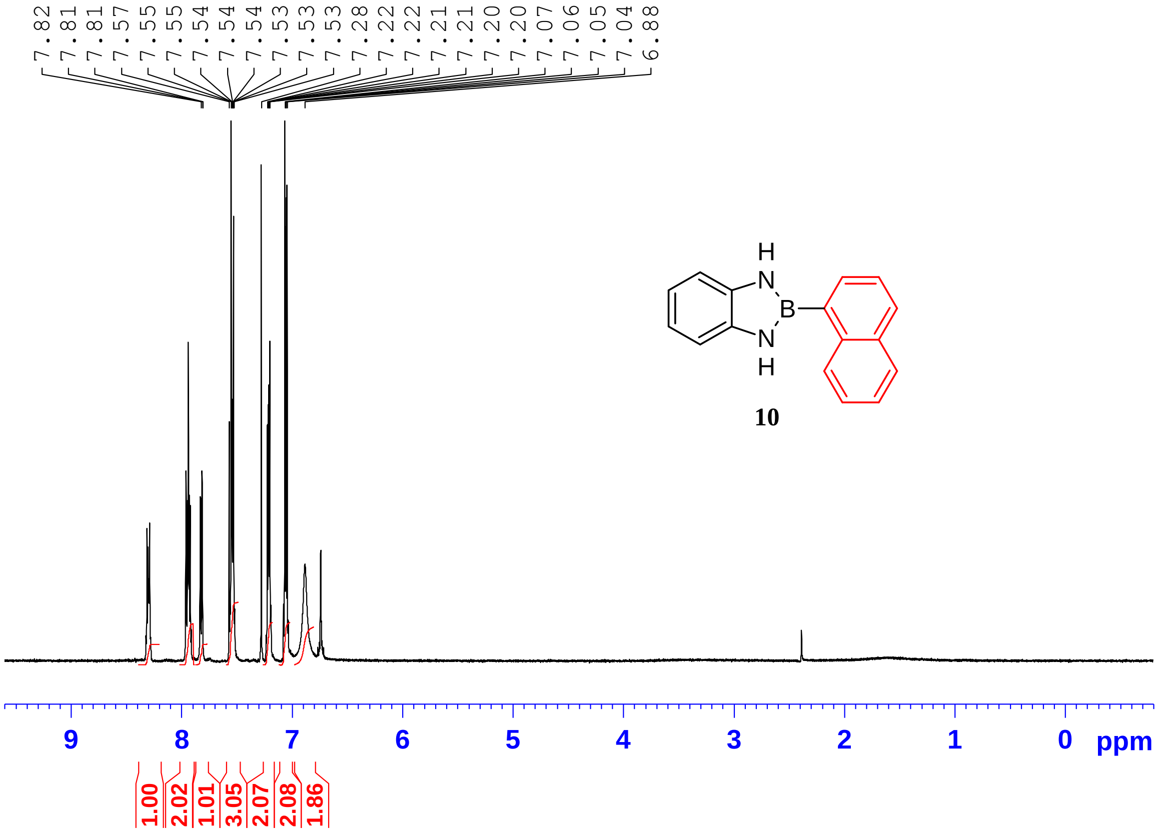
7.82 7.81 7.81 7.57 7.55 7.55 7.54 7.54 7.54 7.53 7.53 7.53 7.28 7.22 7.22 7.21 7.21 7.20 7.20 7.07 7.06 7.05 7.04 6.88
H
N
B
N
H
10
9 8 7 6 5 4 3 2 1 0 ppm
1.00 2.02 1.01 3.05 2.07 2.08 1.86

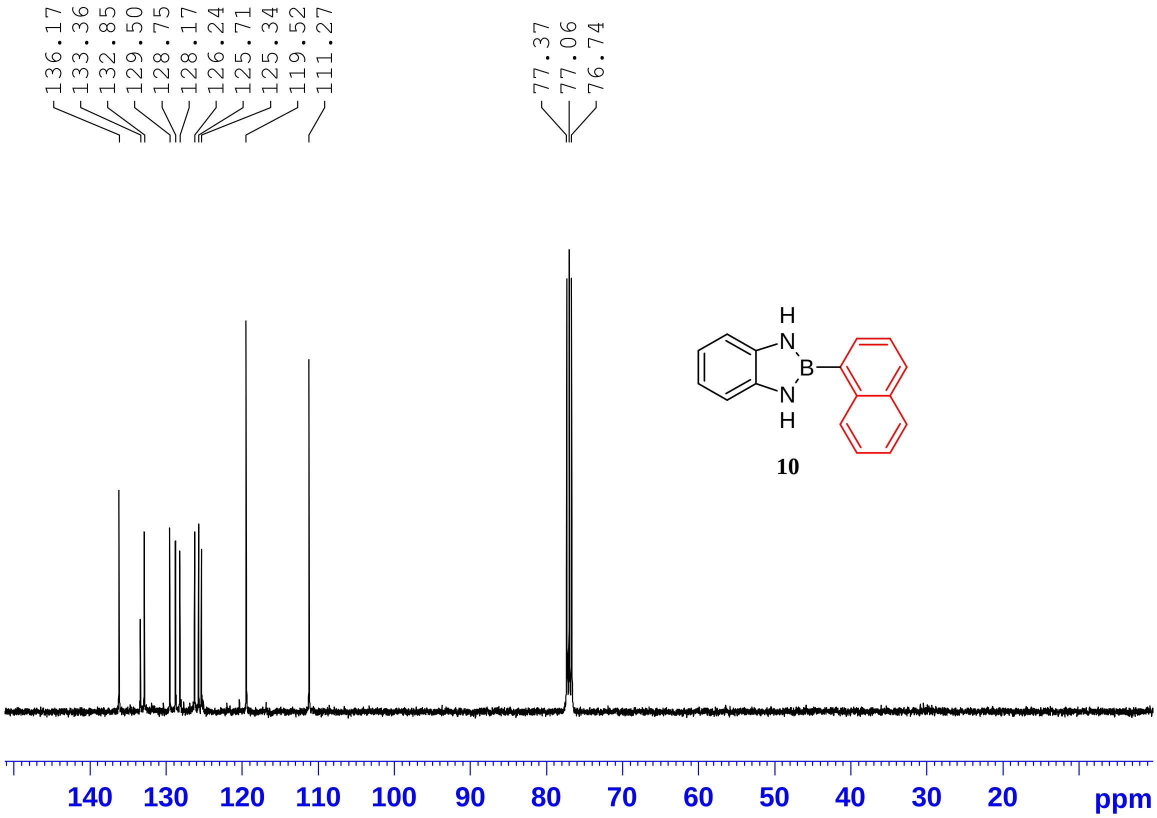
136.17 133.36 132.85 129.50 128.75 128.17 126.24 125.71 125.34 119.52 111.27
77.37 77.06 76.74
H
N
B
N
H
10
140 130 120 110 100 90 80 70 60 50 40 30 20 ppm

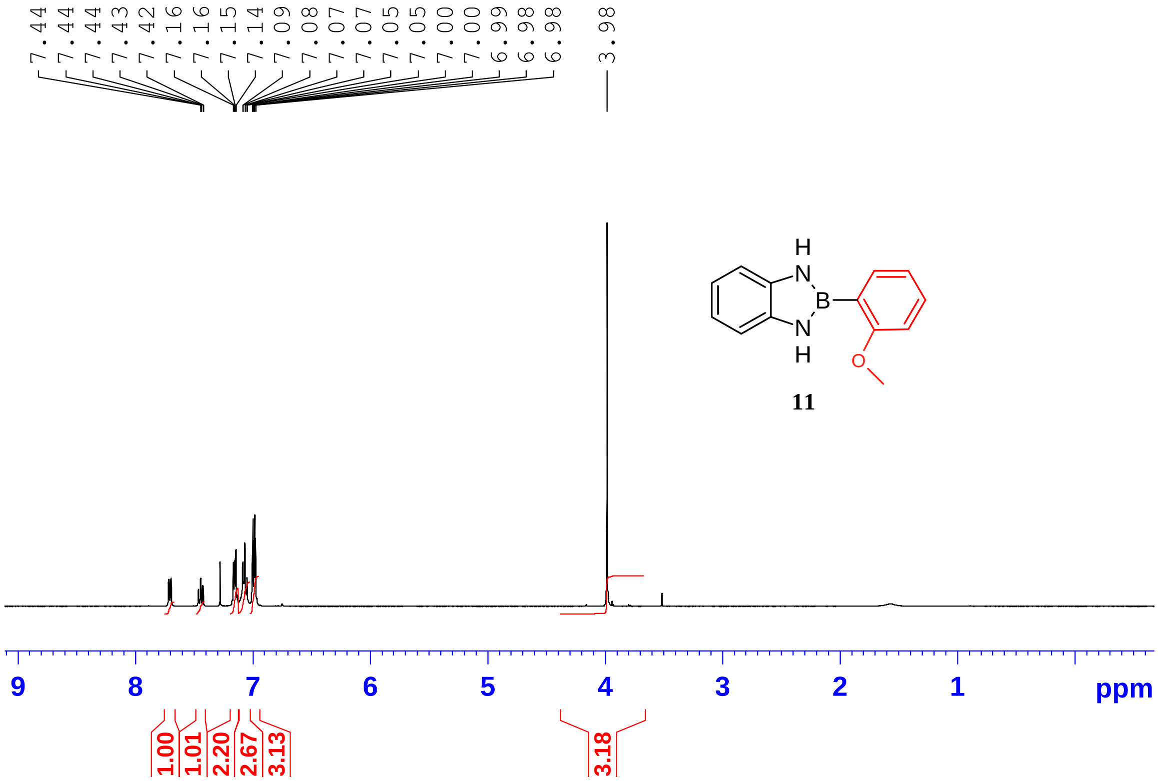
7.44
7.44
7.44
7.43
7.42
7.16
7.16
7.15
7.14
7.09
7.08
7.07
7.07
7.05
7.05
7.00
7.00
6.99
6.98
6.98
3.98
H
N
B
N
H
O
11
9
8
7
6
5
4
3
2
1
ppm
1.00
1.01
2.20
2.67
3.13
3.18

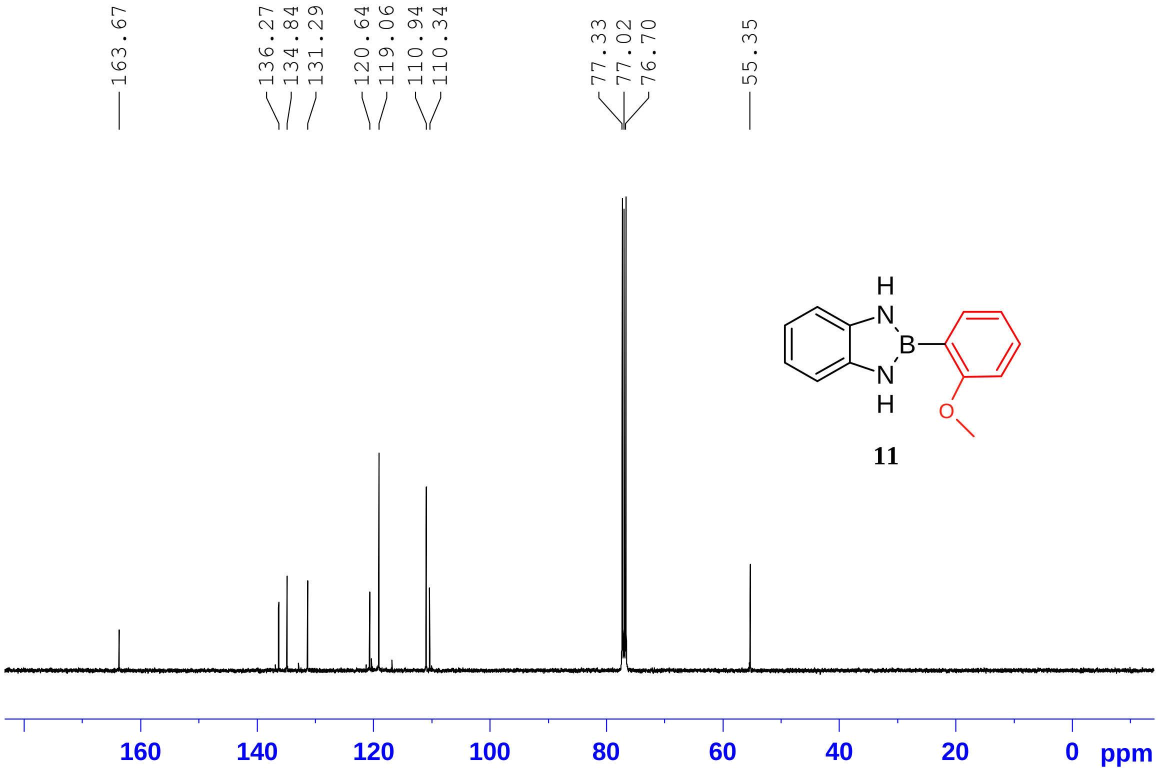
163.67
136.27
134.84
131.29
120.64
119.06
110.94
110.34
77.33
77.02
76.70
55.35
H
N
B
N
H
O
11
160
140
120
100
80
60
40
20
0
ppm

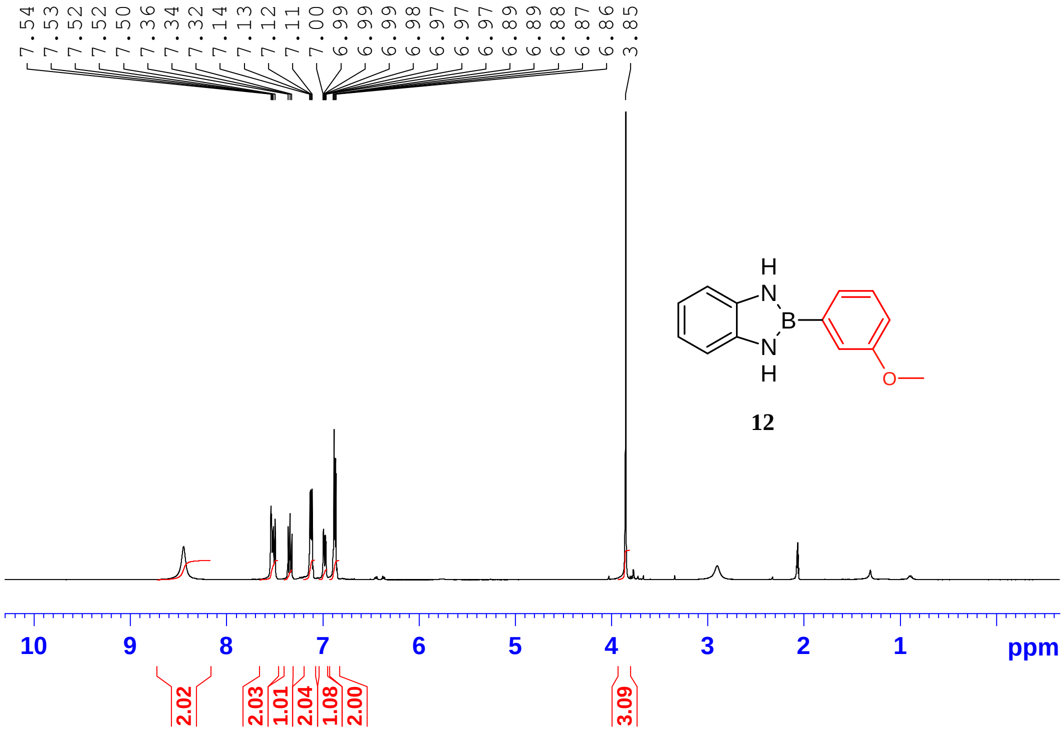
7.54 7.53 7.52 7.52 7.50 7.36 7.34 7.32 7.14 7.13 7.12 7.11 7.00 6.99 6.99 6.99 6.98 6.97 6.97 6.97 6.89 6.89 6.88 6.87 6.86 3.85
H
N
B
N
H
O
12
10 9 8 7 6 5 4 3 2 1 ppm
2.02 2.03 1.01 2.04 1.08 2.00 3.09

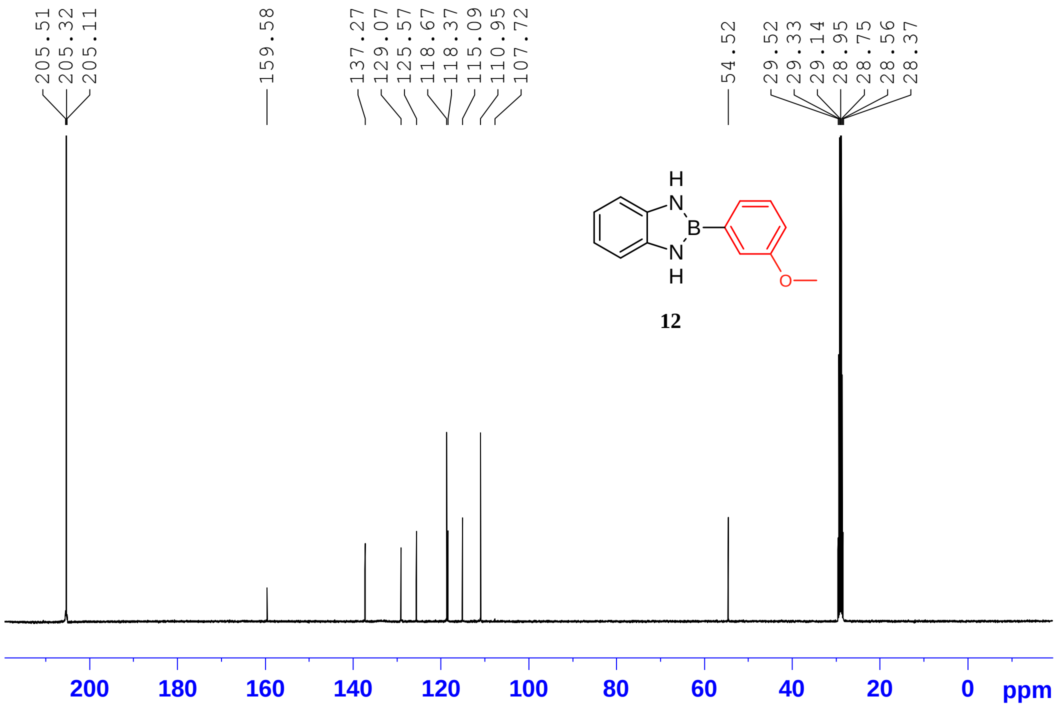
205.51 205.32 205.11 159.58 137.27 129.07 125.57 118.67 118.37 115.09 110.95 107.72 54.52 29.52 29.33 29.14 28.95 28.75 28.56 28.37
H
N
B
N
H
O
12
200 180 160 140 120 100 80 60 40 20 0 ppm

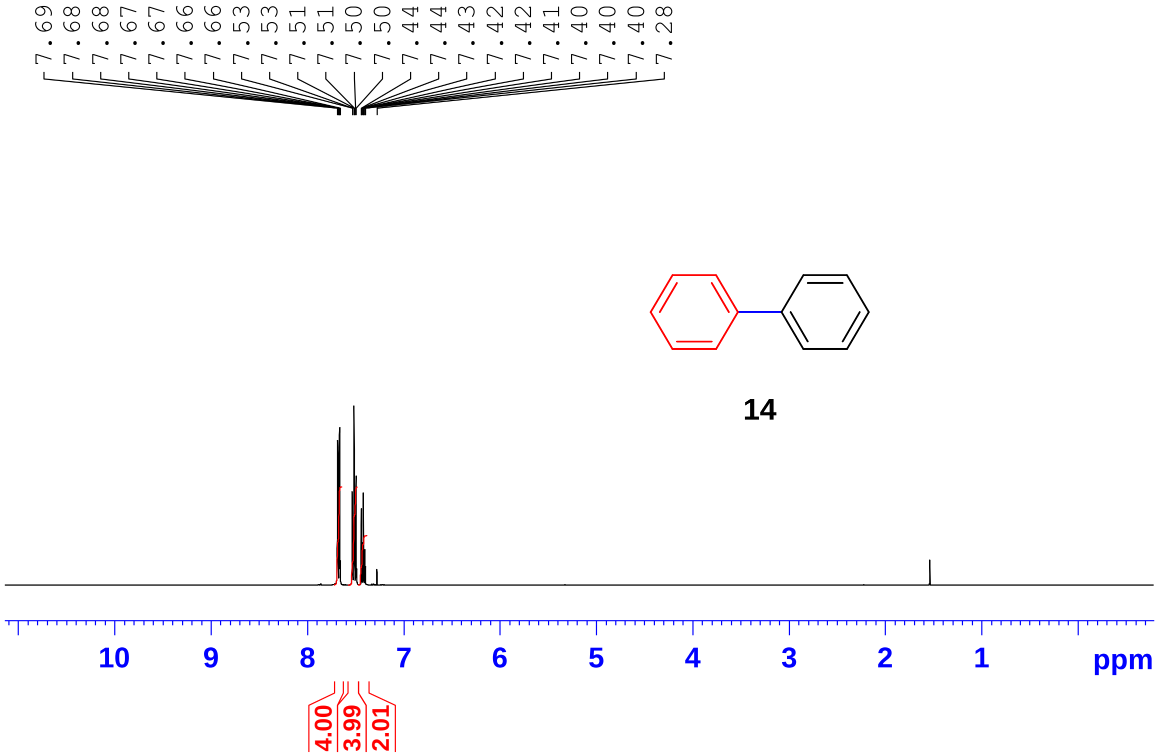
7.69
7.68
7.68
7.67
7.67
7.66
7.66
7.53
7.53
7.51
7.51
7.50
7.50
7.44
7.44
7.43
7.42
7.42
7.41
7.40
7.40
7.40
7.28
14
10
9
8
7
6
5
4
3
2
1
ppm
4.00
3.99
2.01

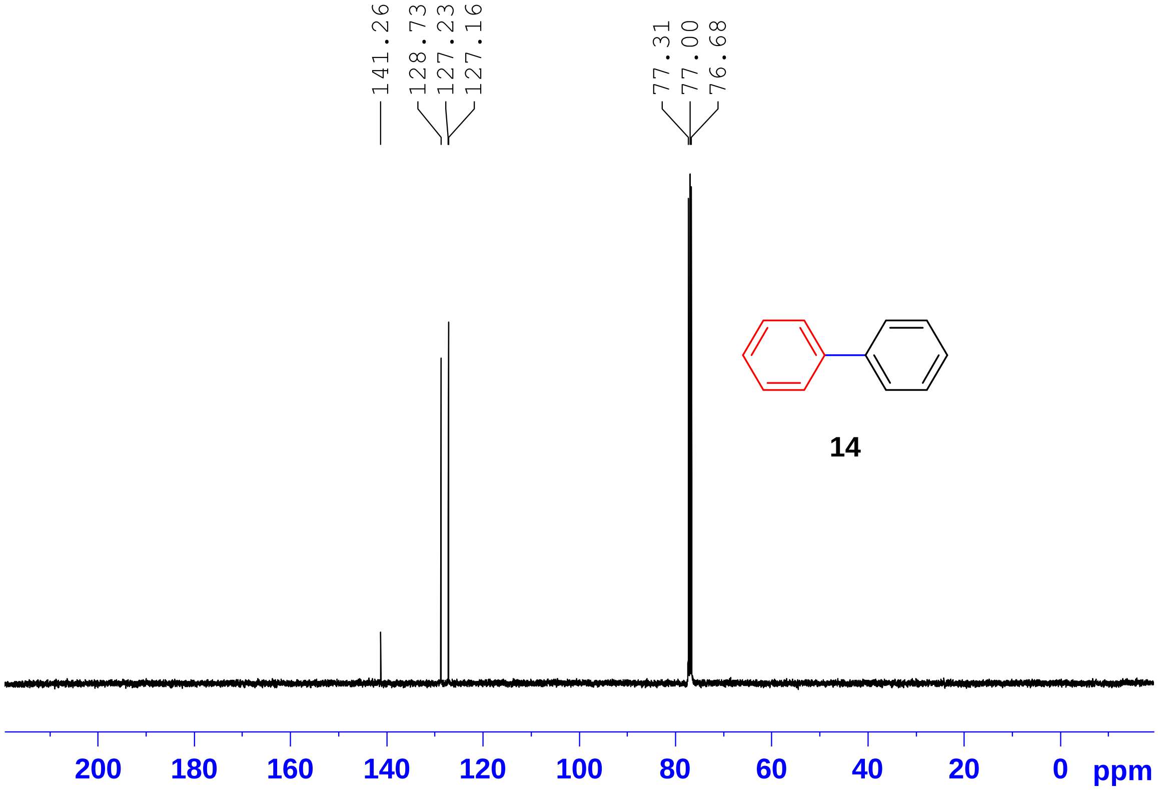
141.26
128.73
127.23
127.16
77.31
77.00
76.68
14
200
180
160
140
120
100
80
60
40
20
0
ppm

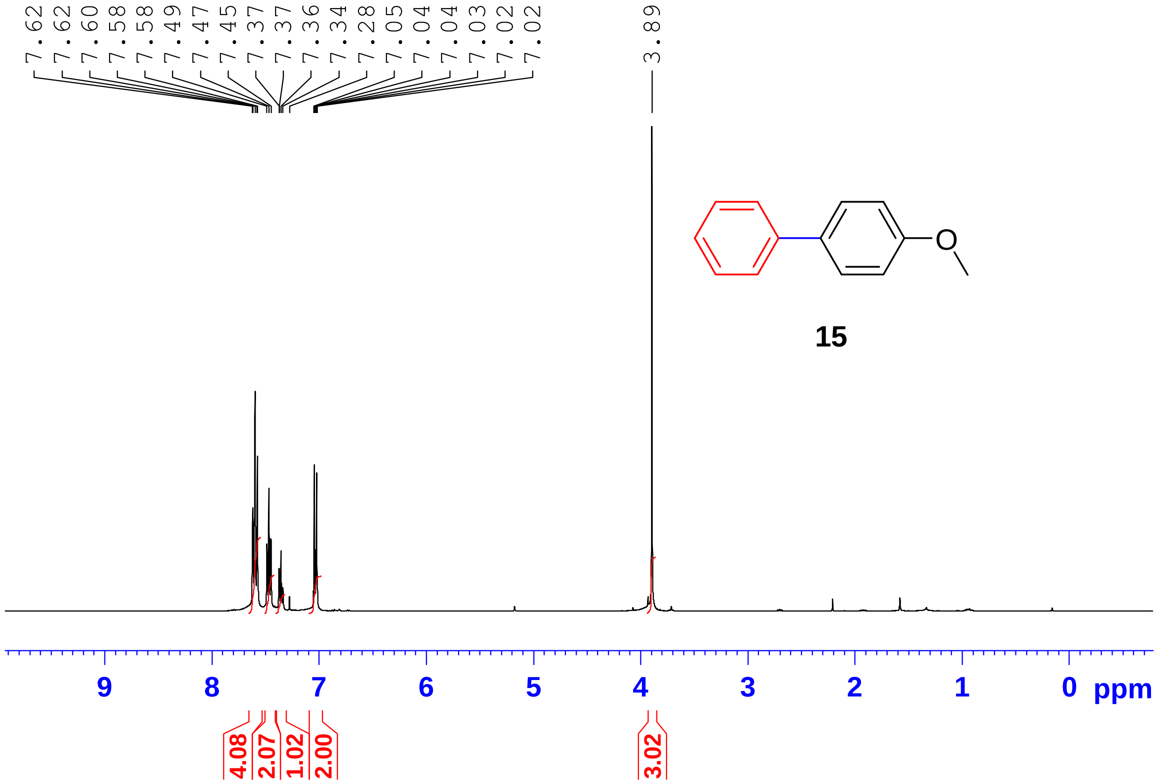
7.62
7.62
7.60
7.58
7.58
7.49
7.47
7.45
7.37
7.37
7.36
7.34
7.28
7.05
7.04
7.04
7.03
7.02
7.02
3.89
O
15
9
8
7
6
5
4
3
2
1
0
ppm
4.08
2.07
1.02
2.00
3.02

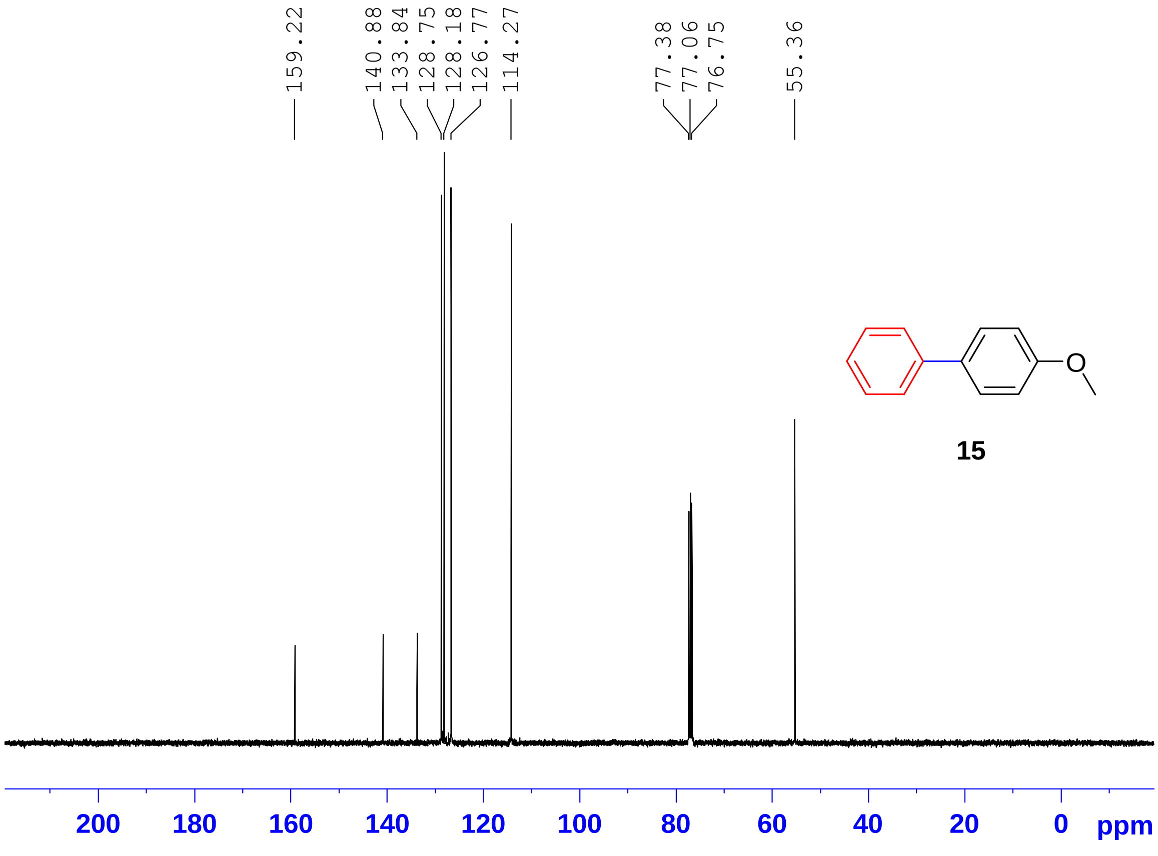
159.22
140.88
133.84
128.75
128.18
126.77
114.27
77.38
77.06
76.75
55.36
O
15
200
180
160
140
120
100
80
60
40
20
0
ppm

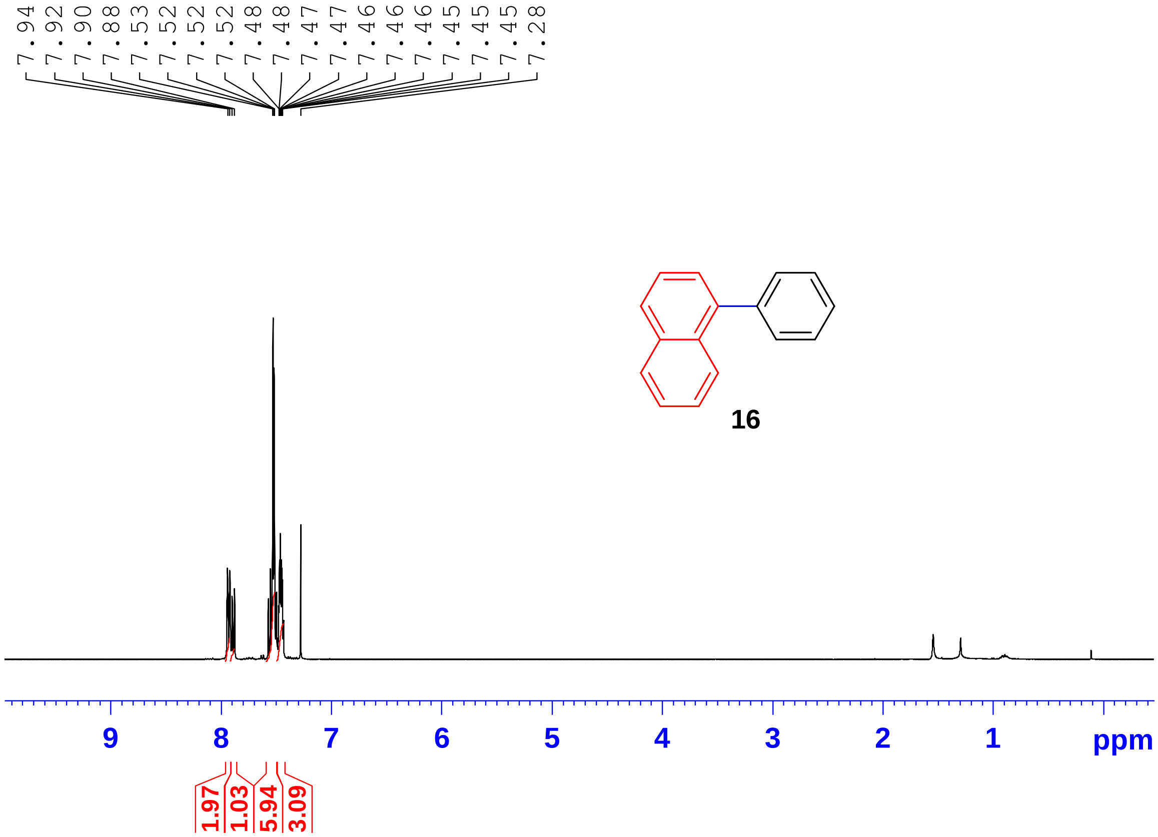
7.94
7.92
7.90
7.88
7.53
7.52
7.52
7.52
7.48
7.48
7.47
7.47
7.46
7.46
7.46
7.45
7.45
7.45
7.28
16
9
8
7
6
5
4
3
2
1
ppm
1.97
1.03
5.94
3.09

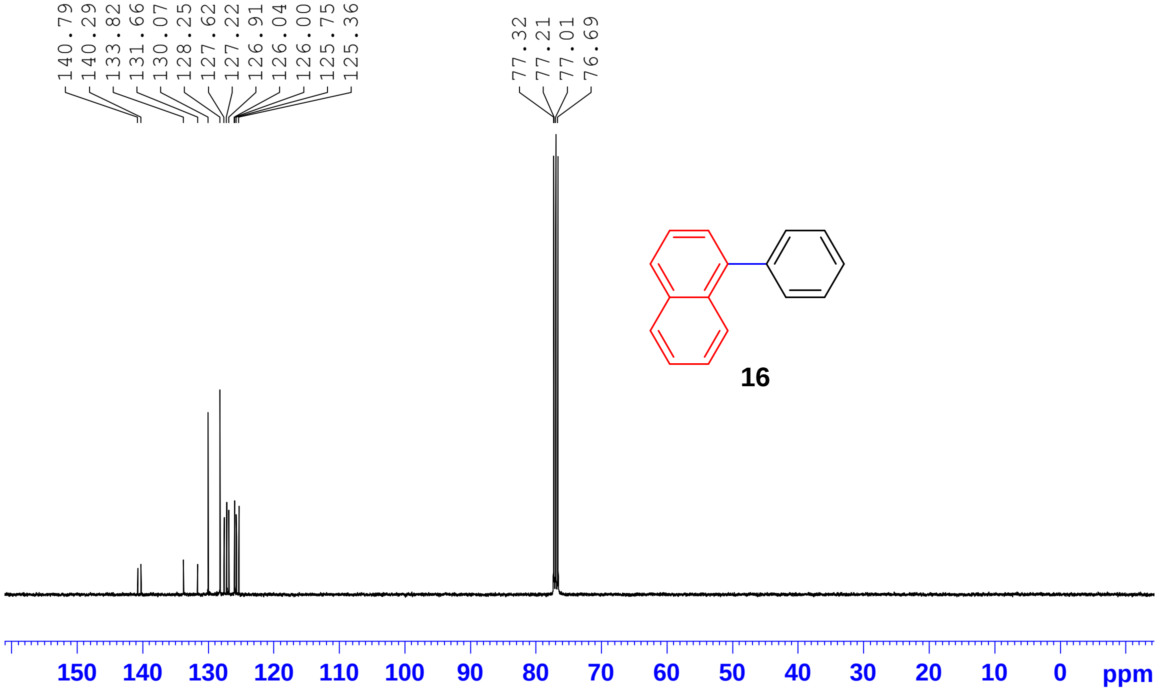
140.79
140.29
133.82
131.66
130.07
128.25
127.62
127.22
126.91
126.04
126.00
125.75
125.36
77.32
77.21
77.01
76.69
16
150
140
130
120
110
100
90
80
70
60
50
40
30
20
10
0
ppm

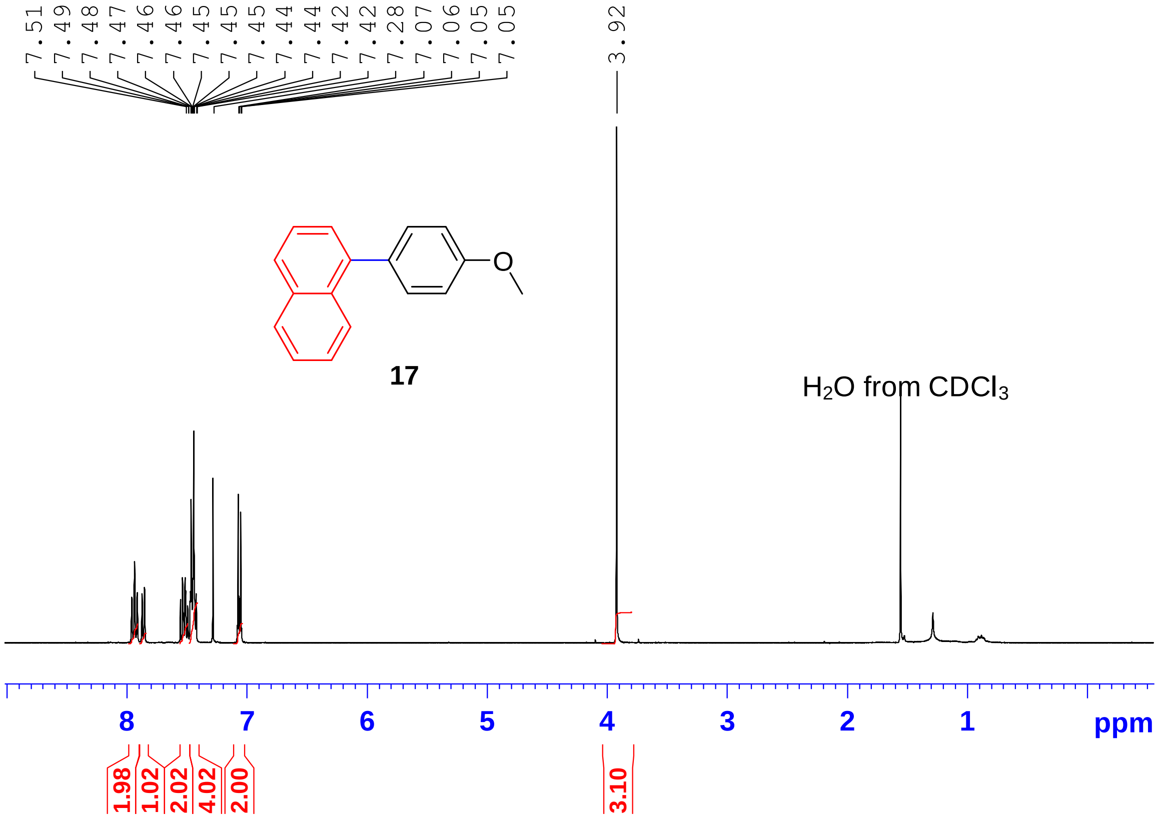

7.51 7.49 7.48 7.47 7.46 7.46 7.45 7.45 7.45 7.44 7.44 7.42 7.42 7.28 7.07 7.06 7.05 7.05
3.92
17
H2O from CDCl3
8 7 6 5 4 3 2 1 ppm
1.98 1.02 2.02 4.02 2.00 3.10

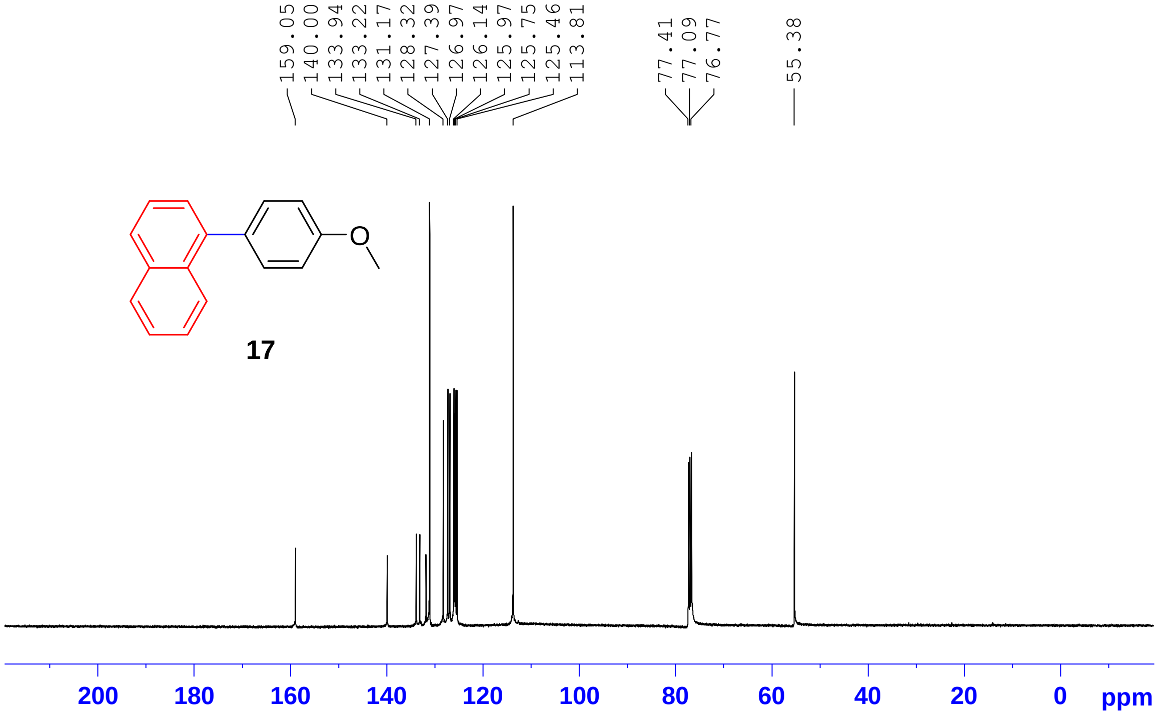

159.05 140.00 133.94 133.22 131.17 128.32 127.39 126.97 126.14 125.97 125.75 125.46 113.81
77.41 77.09 76.77
55.38
17
200 180 160 140 120 100 80 60 40 20 0 ppm

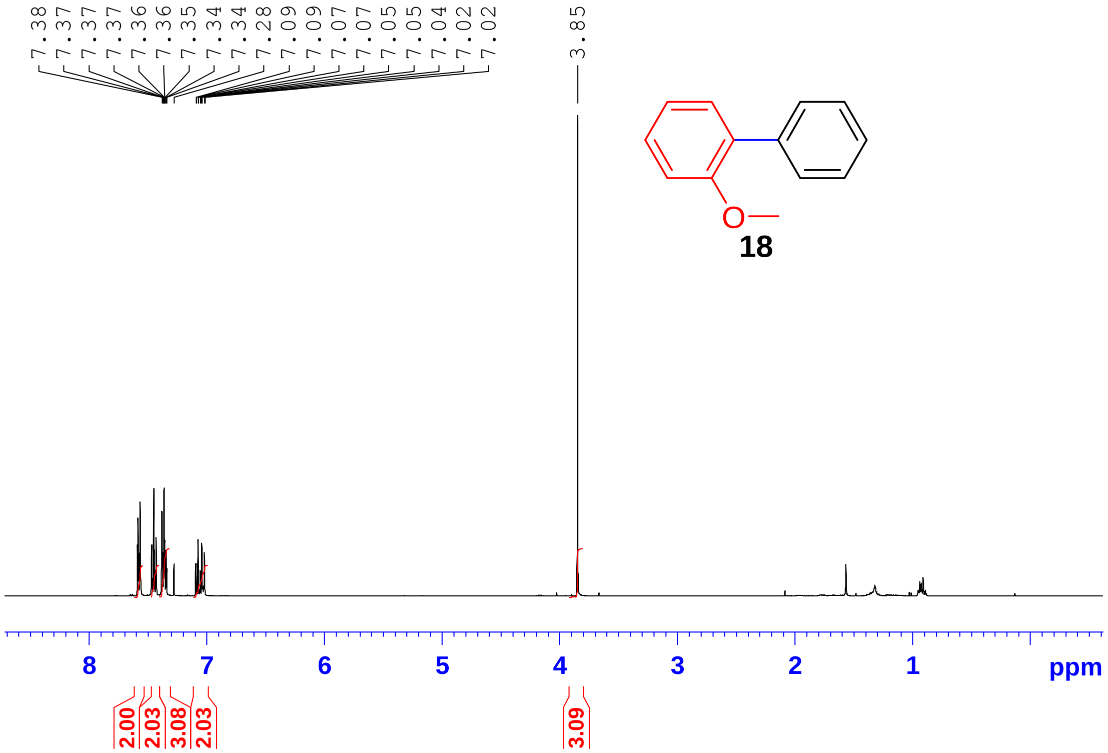
7.38 7.37 7.37 7.37 7.36 7.36 7.35 7.34 7.34 7.28 7.09 7.09 7.07 7.07 7.05 7.05 7.04 7.02 7.02
3.85
18
8 7 6 5 4 3 2 1 ppm
2.00 2.03 3.08 2.03
3.09

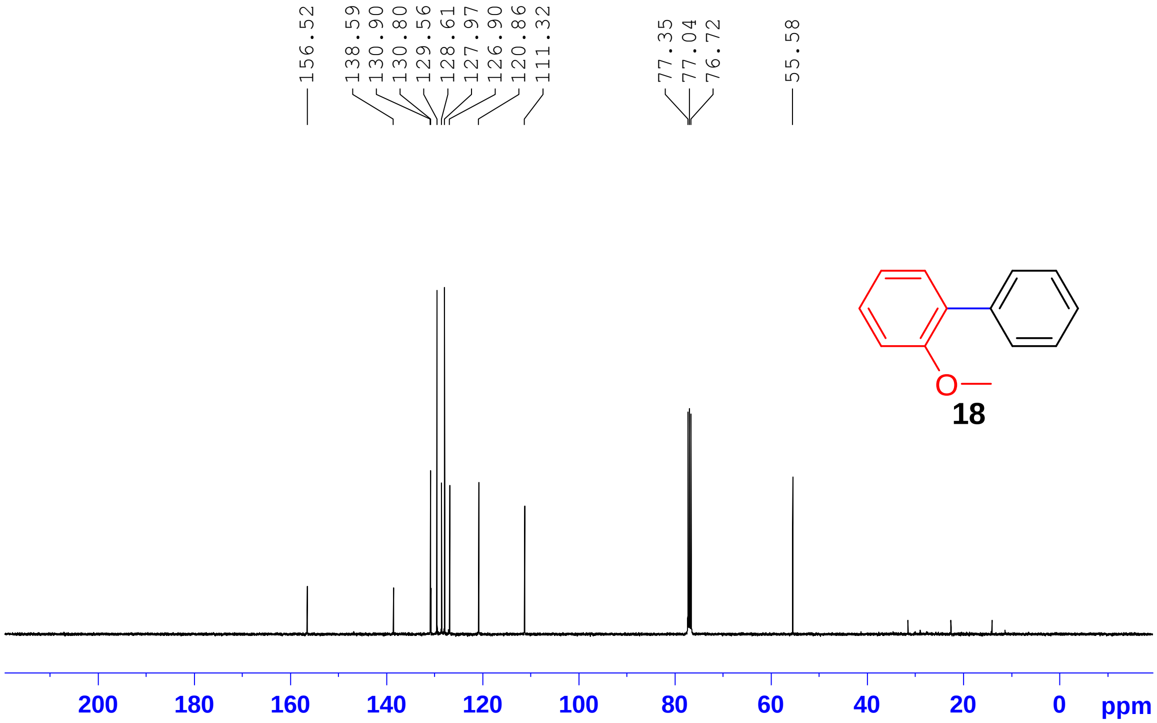
156.52
138.59 130.90 130.80 129.56 128.61 127.97 126.90 120.86 111.32
77.35 77.04 76.72
55.58
18
200 180 160 140 120 100 80 60 40 20 0 ppm

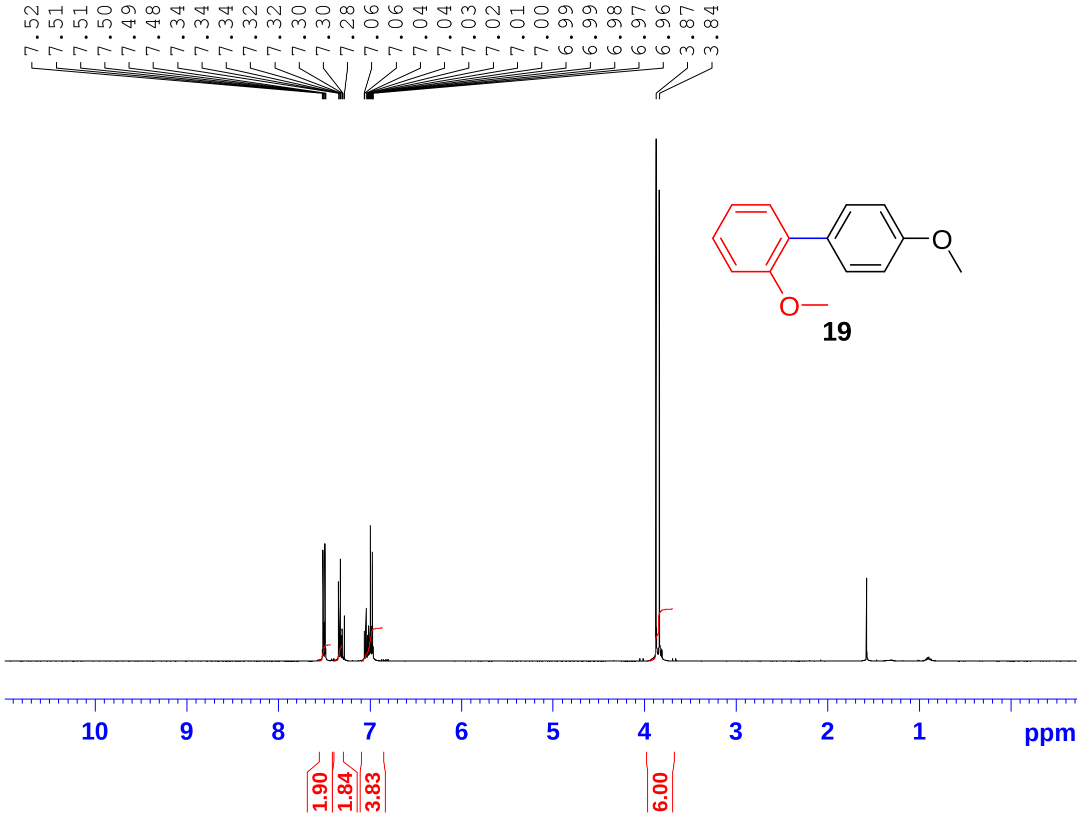
7.52 7.51 7.51 7.50 7.49 7.48 7.34 7.34 7.34 7.32 7.32 7.30 7.30 7.28 7.06 7.06 7.04 7.04 7.03 7.02 7.01 7.00 6.99 6.99 6.98 6.97 6.96 3.87 3.84
O
O
19
10 9 8 7 6 5 4 3 2 1 ppm
1.90 1.84 3.83 6.00

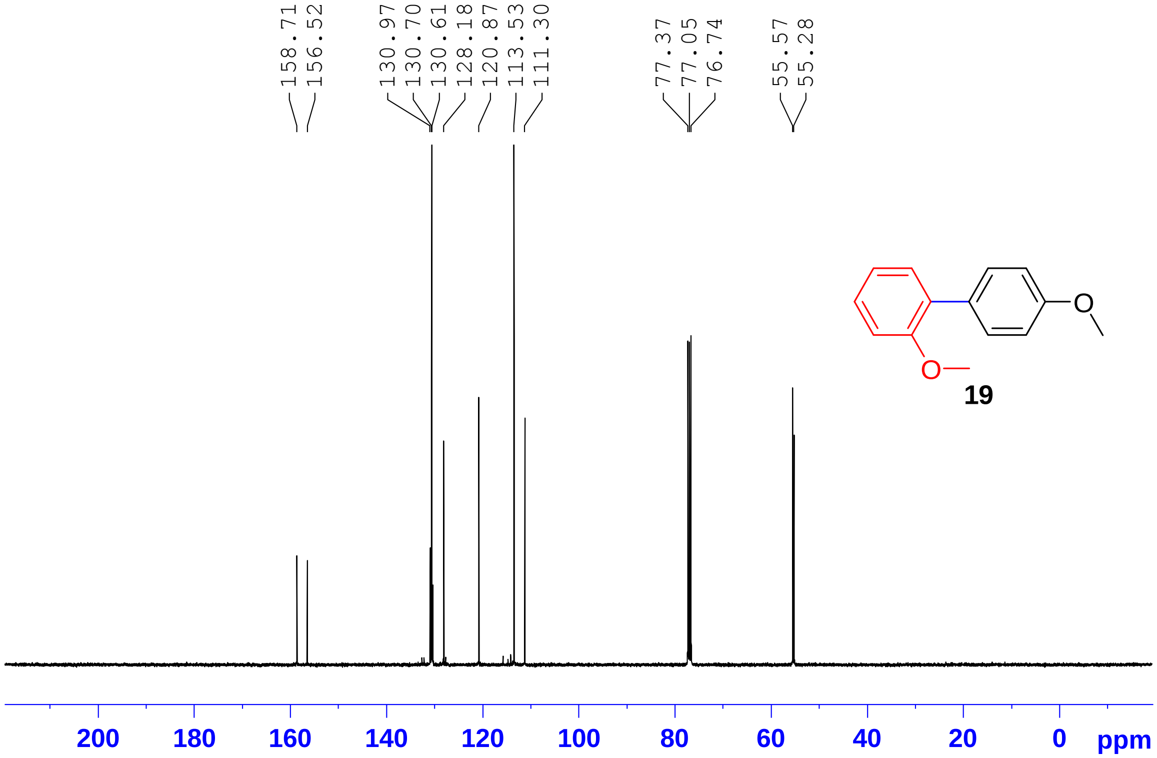
158.71 156.52 130.97 130.70 130.61 128.18 120.87 113.53 111.30 77.37 77.05 76.74 55.57 55.28
O
O
19
200 180 160 140 120 100 80 60 40 20 0 ppm

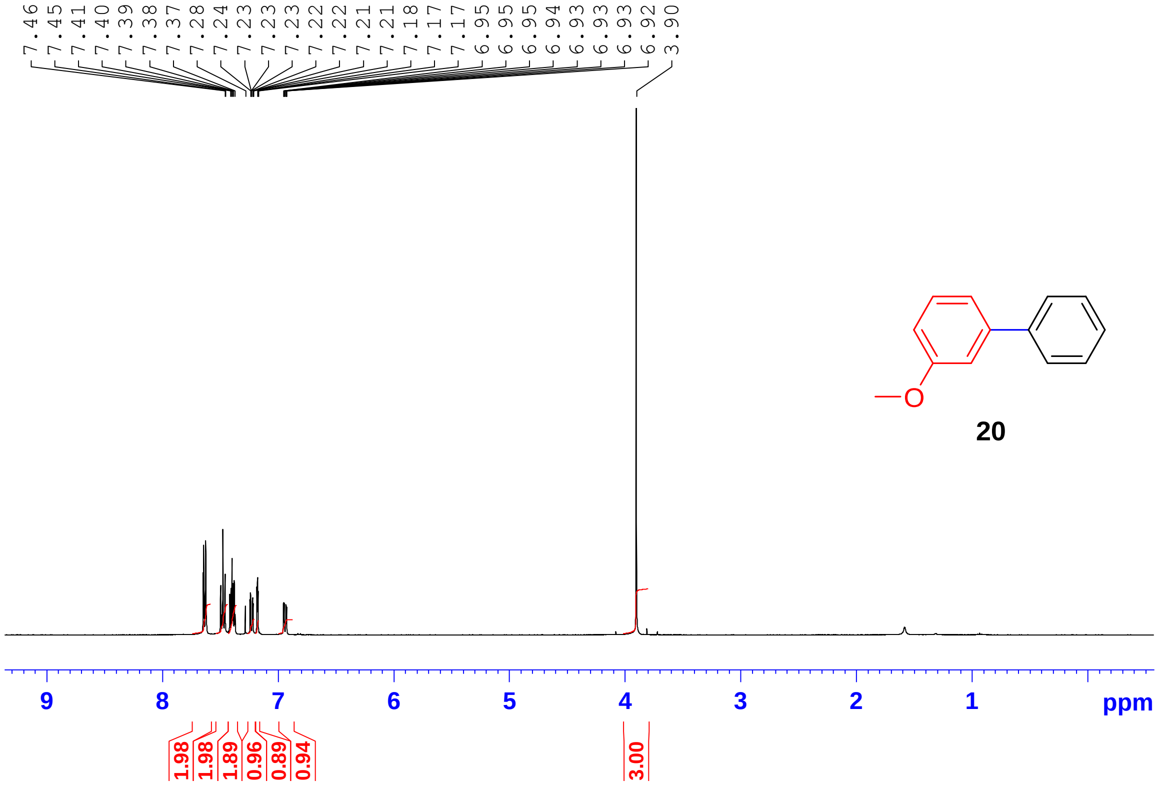
7.46
7.45
7.41
7.40
7.39
7.38
7.37
7.28
7.24
7.23
7.23
7.23
7.22
7.22
7.21
7.21
7.18
7.17
7.17
6.95
6.95
6.95
6.94
6.93
6.93
6.93
6.92
3.90
20
9
8
7
6
5
4
3
2
1
ppm
1.98
1.98
1.89
0.96
0.89
0.94
3.00

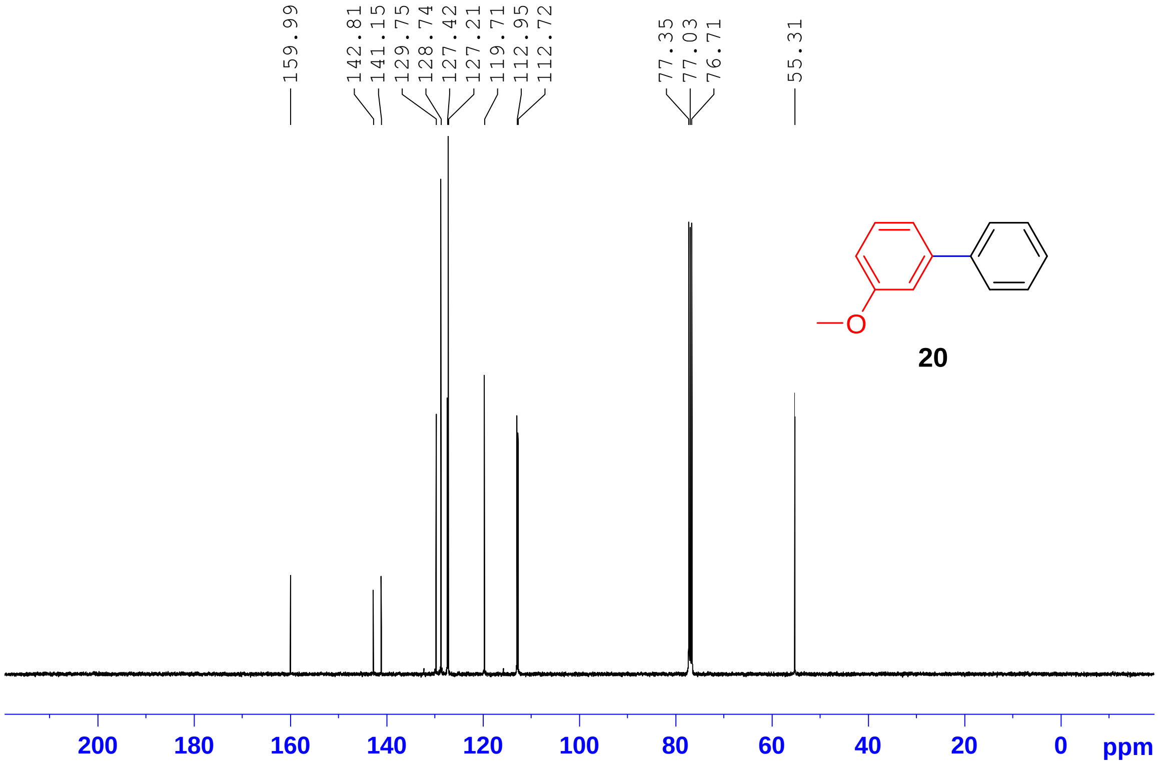
159.99
142.81
141.15
129.75
128.74
127.42
127.21
119.71
112.95
112.72
77.35
77.03
76.71
55.31
20
200
180
160
140
120
100
80
60
40
20
0
ppm

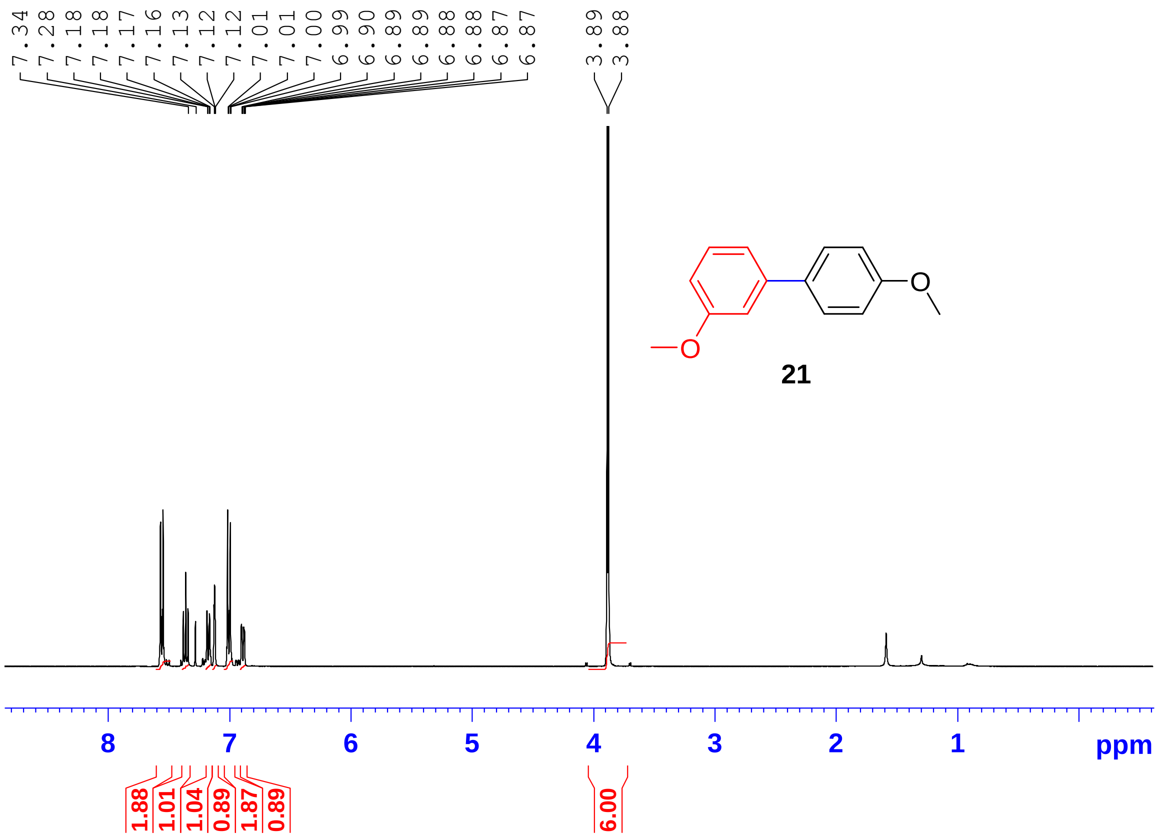
7.34 7.28 7.18 7.18 7.17 7.16 7.13 7.12 7.12 7.01 7.01 7.00 6.99 6.90 6.89 6.89 6.88 6.88 6.87 6.87
3.89 3.88
O
O
21
8
7
6
5
4
3
2
1
ppm
1.88
1.01
1.04
0.89
1.87
0.89
6.00

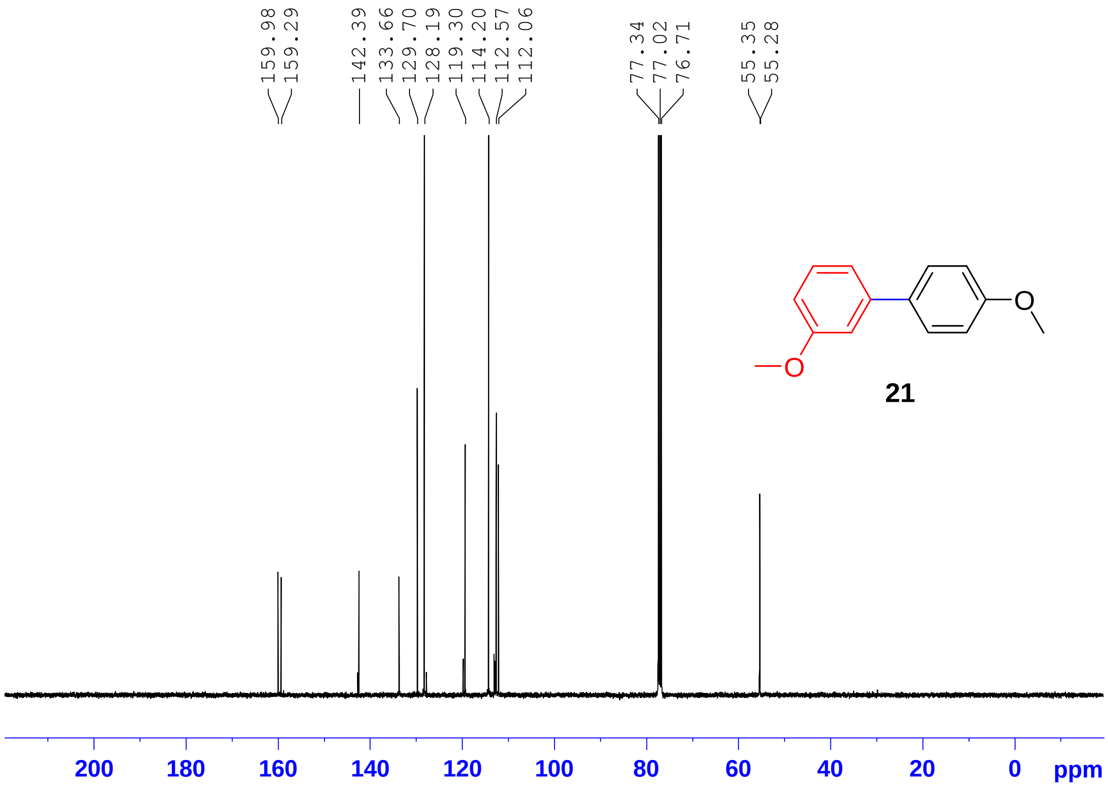
159.98 159.29
142.39 133.66 129.70 128.19 119.30 114.20 112.57 112.06
77.34 77.02 76.71
55.35 55.28
O
O
21
200
180
160
140
120
100
80
60
40
20
0
ppm

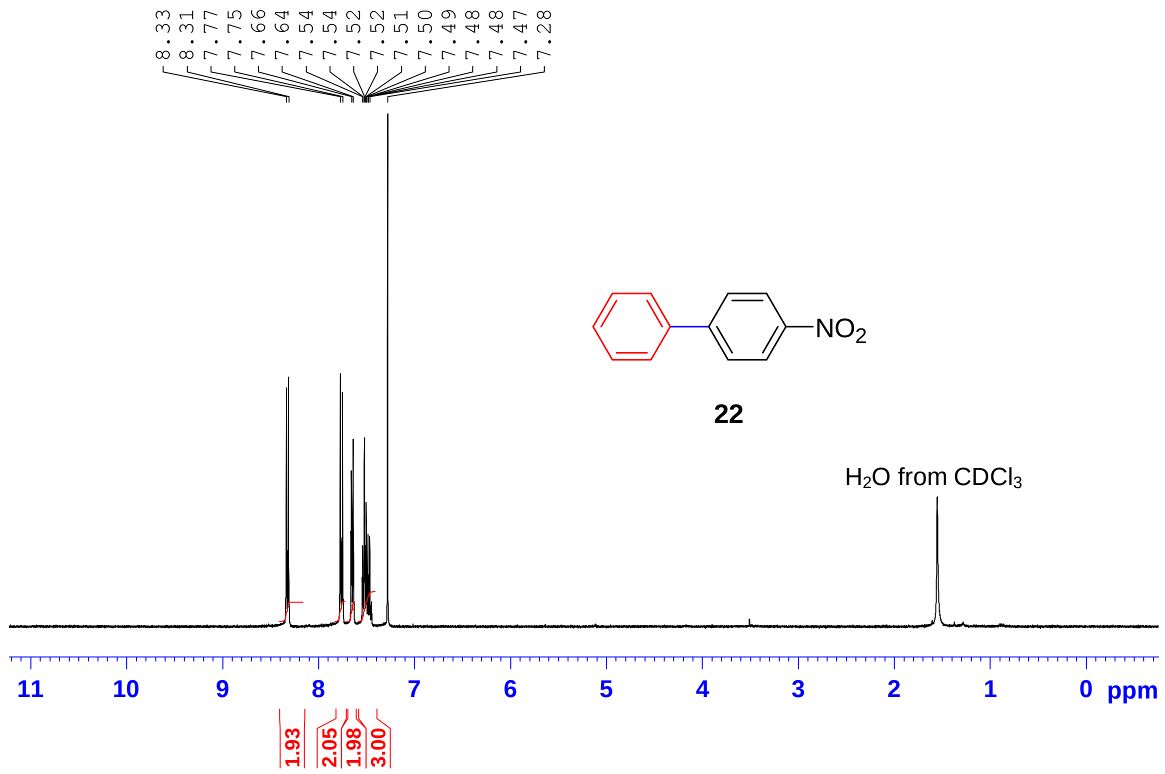
8.33
8.31
7.77
7.75
7.66
7.64
7.54
7.54
7.52
7.52
7.51
7.50
7.49
7.48
7.48
7.47
7.28
NO2
22
H2O from CDCl3
11 10 9 8 7 6 5 4 3 2 1 0 ppm
1.93
2.05
1.98
3.00

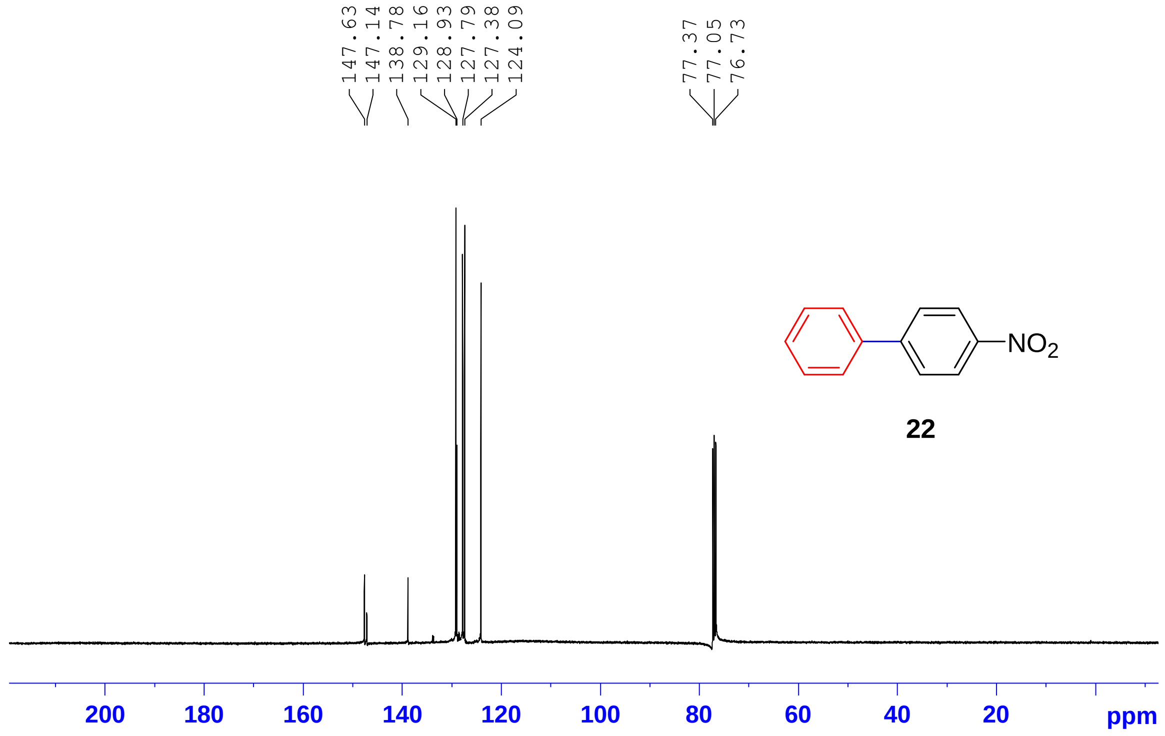
147.63
147.14
138.78
129.16
128.93
127.79
127.38
124.09
77.37
77.05
76.73
NO2
22
200 180 160 140 120 100 80 60 40 20 ppm

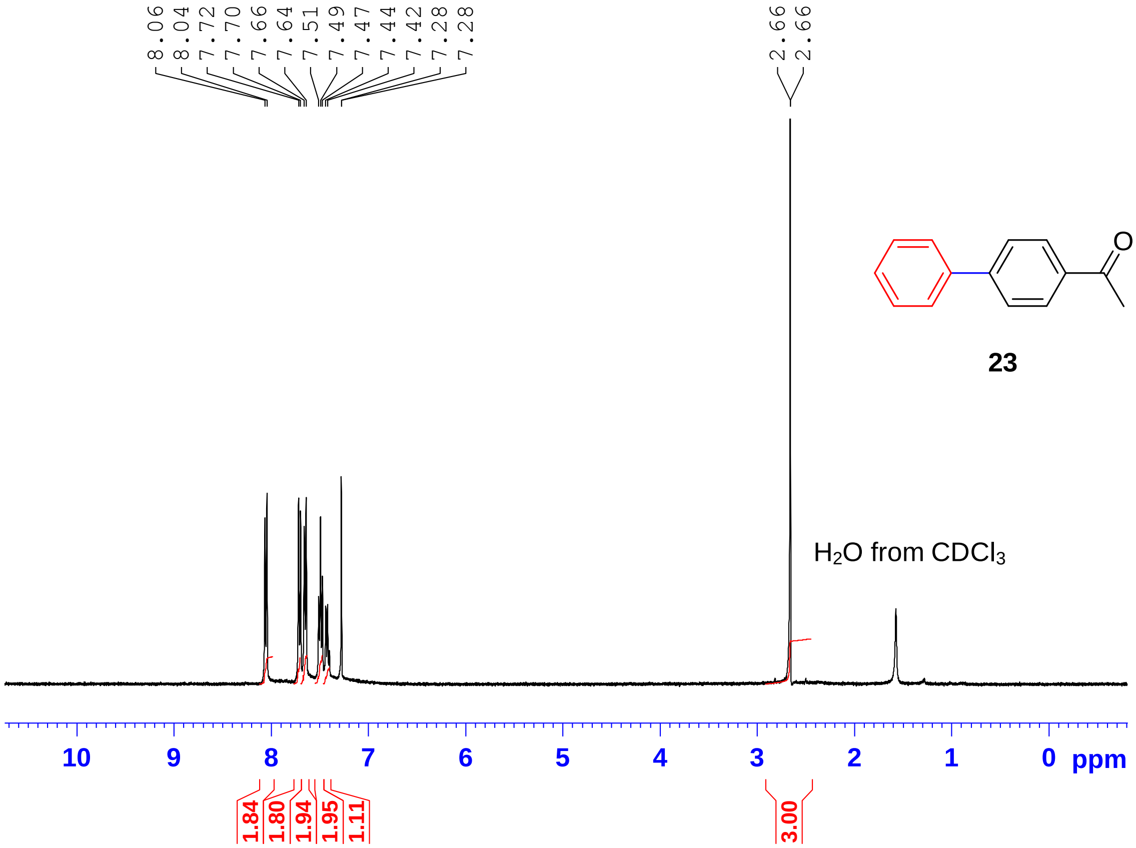

8.06
8.04
7.72
7.70
7.66
7.64
7.51
7.49
7.47
7.44
7.42
7.28
7.28
2.66
2.66
O
23
H$_2$O from CDCl$_3$
10 9 8 7 6 5 4 3 2 1 0 ppm
1.84
1.80
1.94
1.95
1.11
3.00

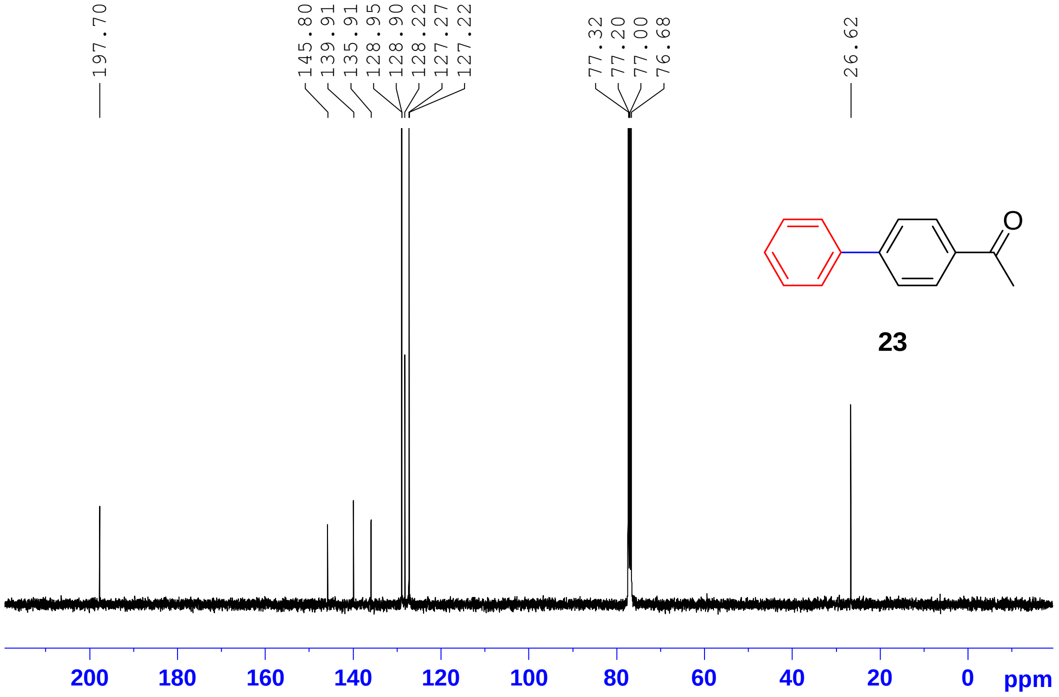

197.70
145.80
139.91
135.91
128.95
128.90
128.22
127.27
127.22
77.32
77.20
77.00
76.68
26.62
O
23
200 180 160 140 120 100 80 60 40 20 0 ppm

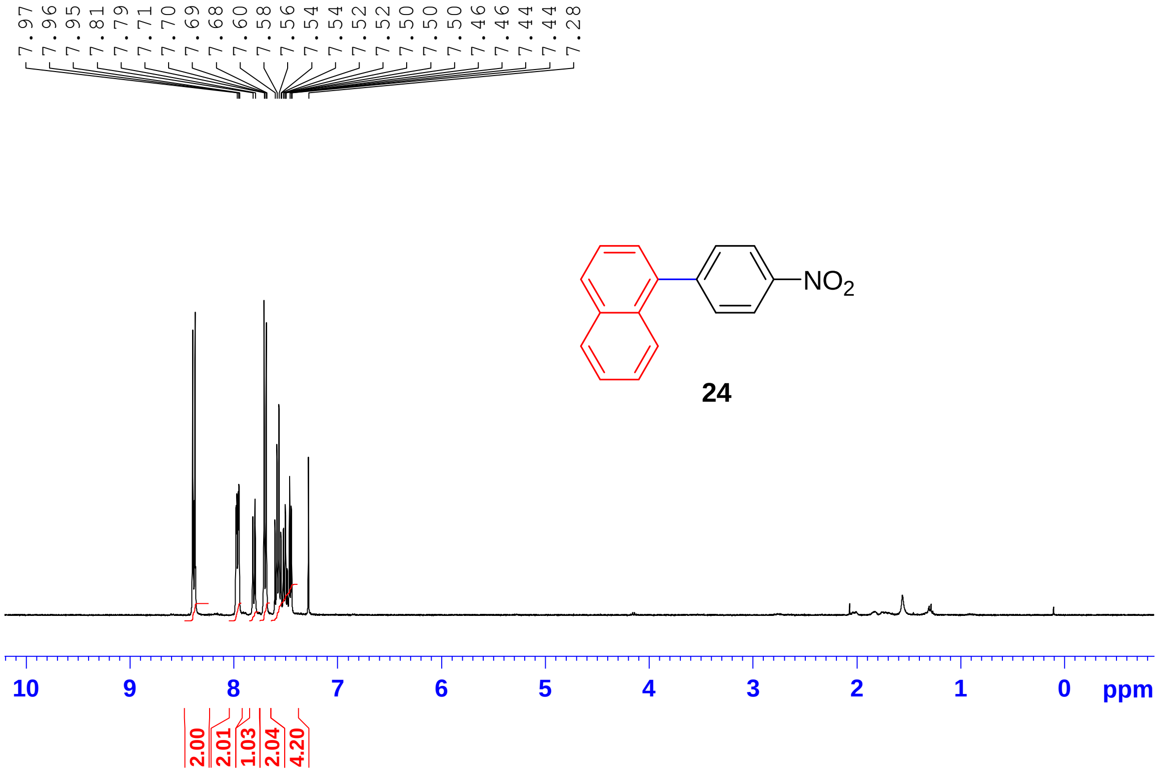
7.97
7.96
7.95
7.81
7.79
7.71
7.70
7.69
7.68
7.60
7.58
7.56
7.54
7.54
7.52
7.52
7.50
7.50
7.50
7.46
7.46
7.44
7.44
7.28
NO_2
24
10 9 8 7 6 5 4 3 2 1 0 ppm
2.00
2.01
1.03
2.04
4.20

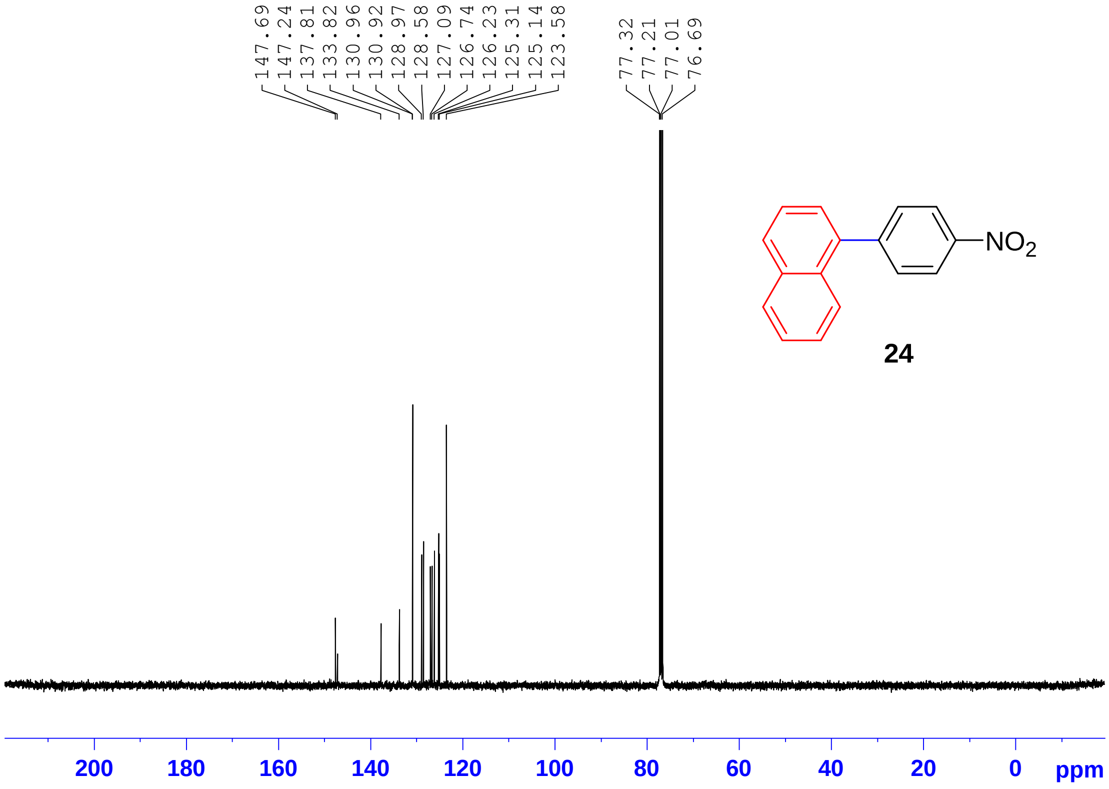
147.69
147.24
137.81
133.82
130.96
130.92
128.97
128.58
127.09
126.74
126.23
125.31
125.14
123.58
77.32
77.21
77.01
76.69
NO_2
24
200 180 160 140 120 100 80 60 40 20 0 ppm

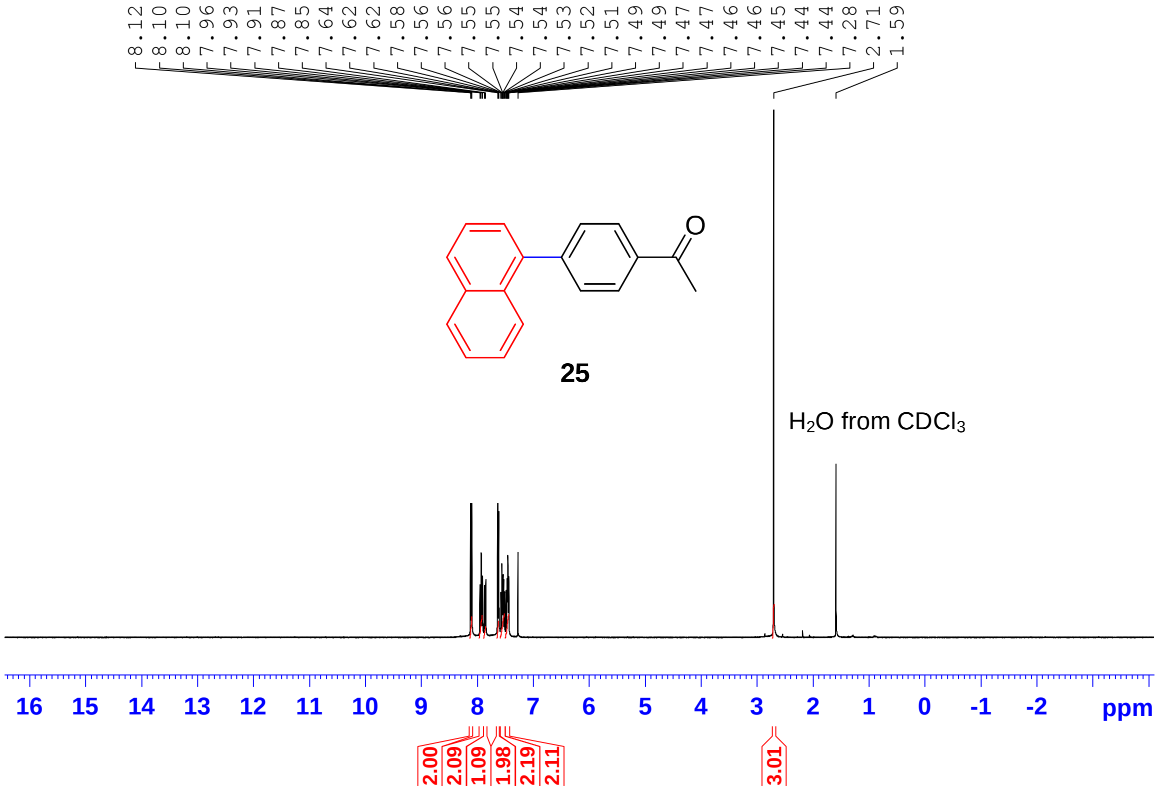
8.12
8.10
8.10
7.96
7.93
7.91
7.87
7.85
7.64
7.62
7.62
7.58
7.56
7.56
7.55
7.55
7.54
7.54
7.53
7.52
7.51
7.49
7.49
7.47
7.47
7.46
7.46
7.45
7.44
7.44
7.28
2.71
1.59
O
25
H2O from CDCl3
16 15 14 13 12 11 10 9 8 7 6 5 4 3 2 1 0 -1 -2 ppm
2.00
2.09
1.09
1.98
2.19
2.11
3.01

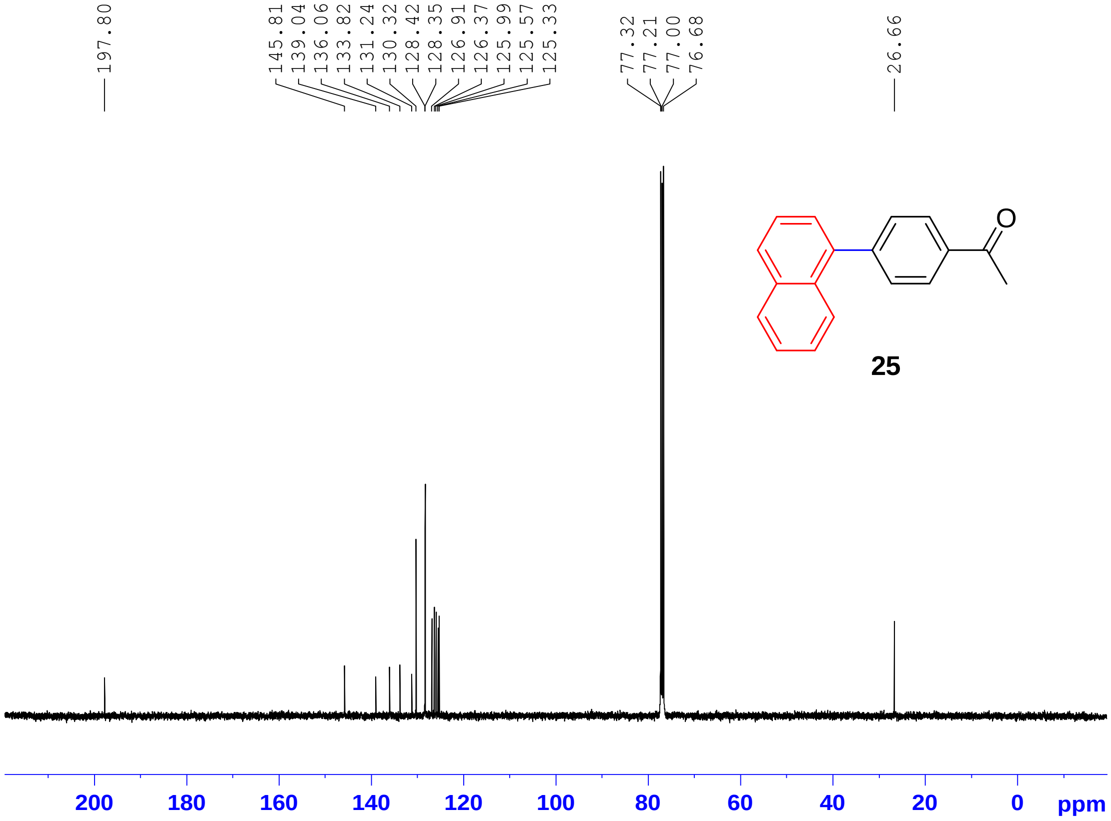
197.80
145.81
139.04
136.06
133.82
131.24
130.32
128.42
128.35
126.91
126.37
125.99
125.57
125.33
77.32
77.21
77.00
76.68
26.66
O
25
200 180 160 140 120 100 80 60 40 20 0 ppm

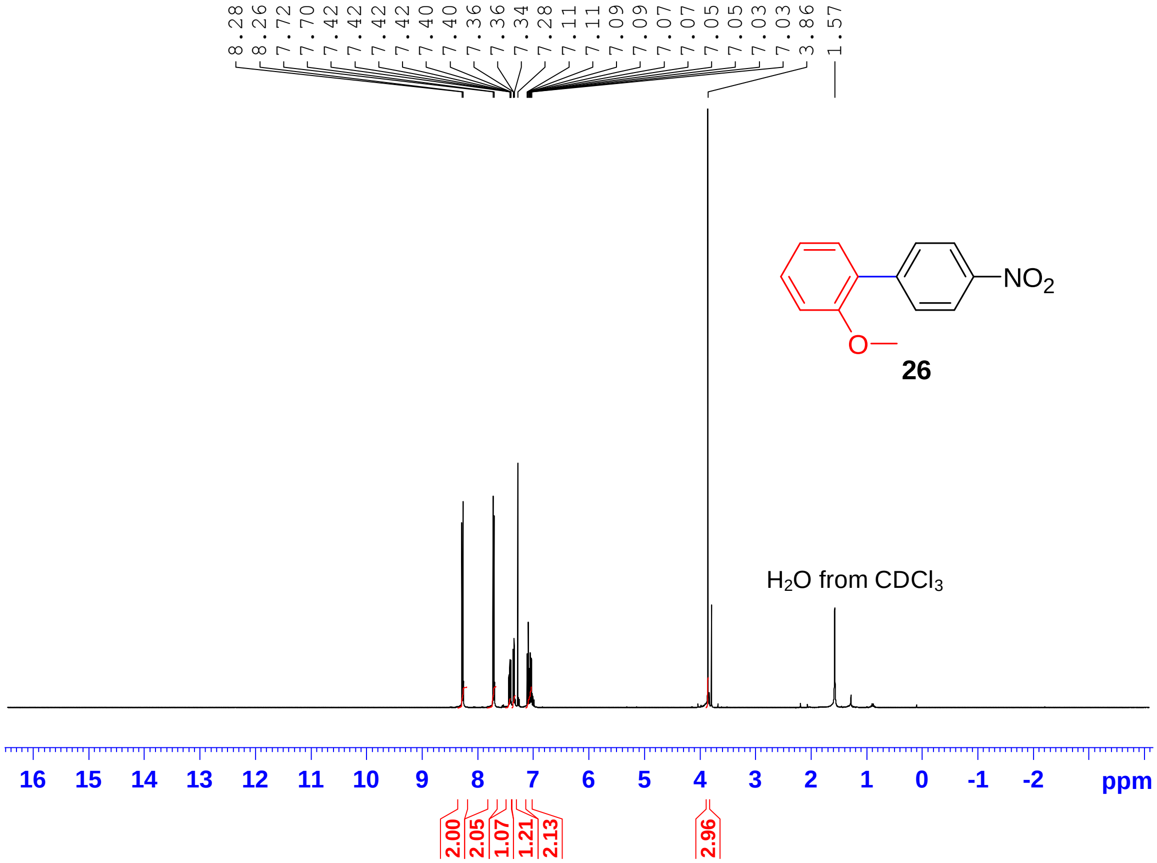
8.28 8.26 7.72 7.70 7.42 7.42 7.42 7.42 7.40 7.40 7.36 7.36 7.34 7.28 7.11 7.11 7.09 7.09 7.07 7.07 7.05 7.05 7.03 7.03 3.86 1.57
NO_2
26
H_2O from $CDCl_3$
16 15 14 13 12 11 10 9 8 7 6 5 4 3 2 1 0 -1 -2 ppm
2.00 2.05 1.07 1.21 2.13 2.96

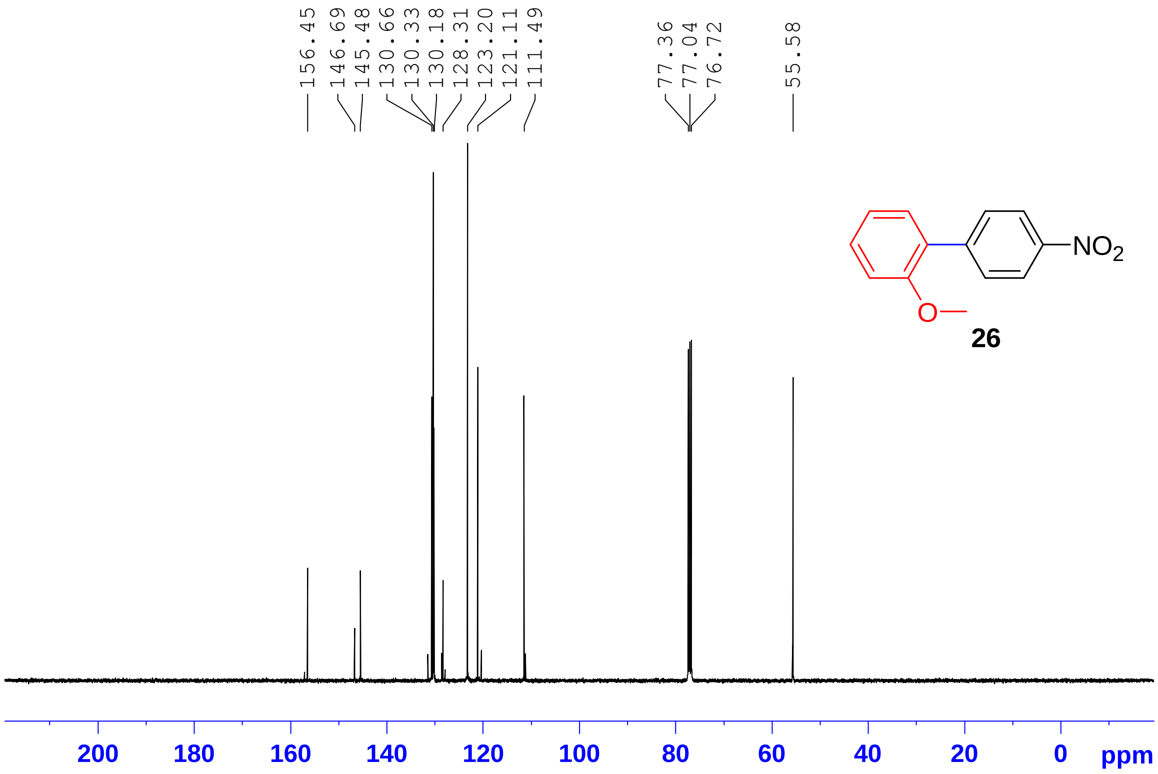
156.45 146.69 145.48 130.66 130.33 130.18 128.31 123.20 121.11 111.49 77.36 77.04 76.72 55.58
NO_2
26
200 180 160 140 120 100 80 60 40 20 0 ppm

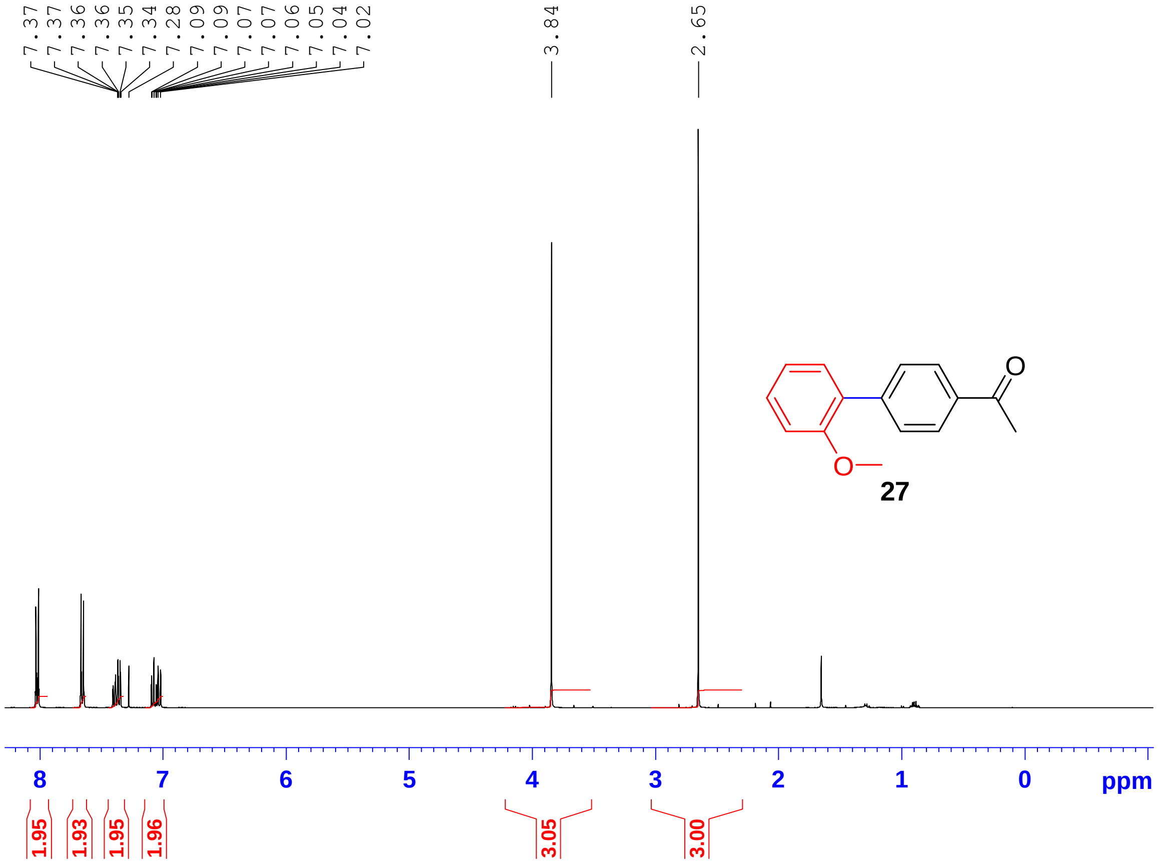
7.37
7.37
7.36
7.36
7.35
7.34
7.28
7.09
7.09
7.07
7.07
7.06
7.05
7.04
7.02
3.84
2.65
O
O
27
8
7
6
5
4
3
2
1
0
ppm
1.95
1.93
1.95
1.96
3.05
3.00

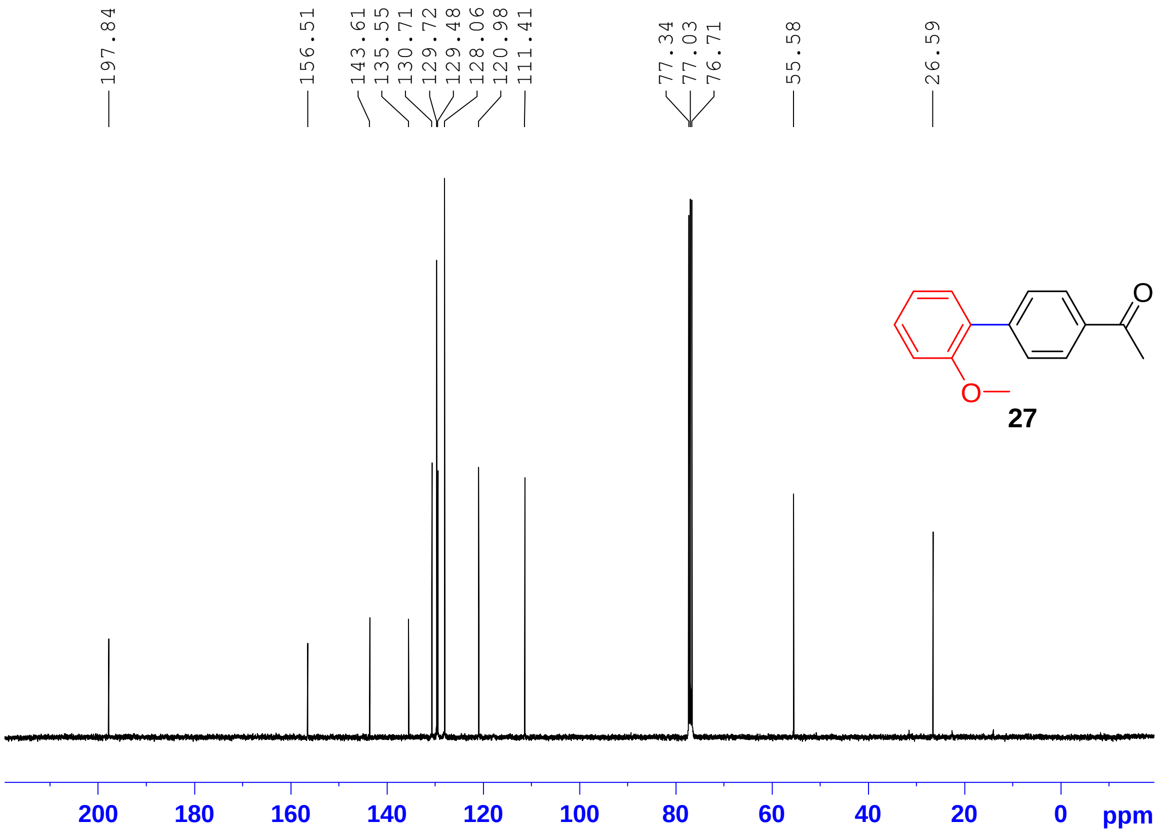
197.84
156.51
143.61
135.55
130.71
129.72
129.48
128.06
120.98
111.41
77.34
77.03
76.71
55.58
26.59
O
O
27
200
180
160
140
120
100
80
60
40
20
0
ppm

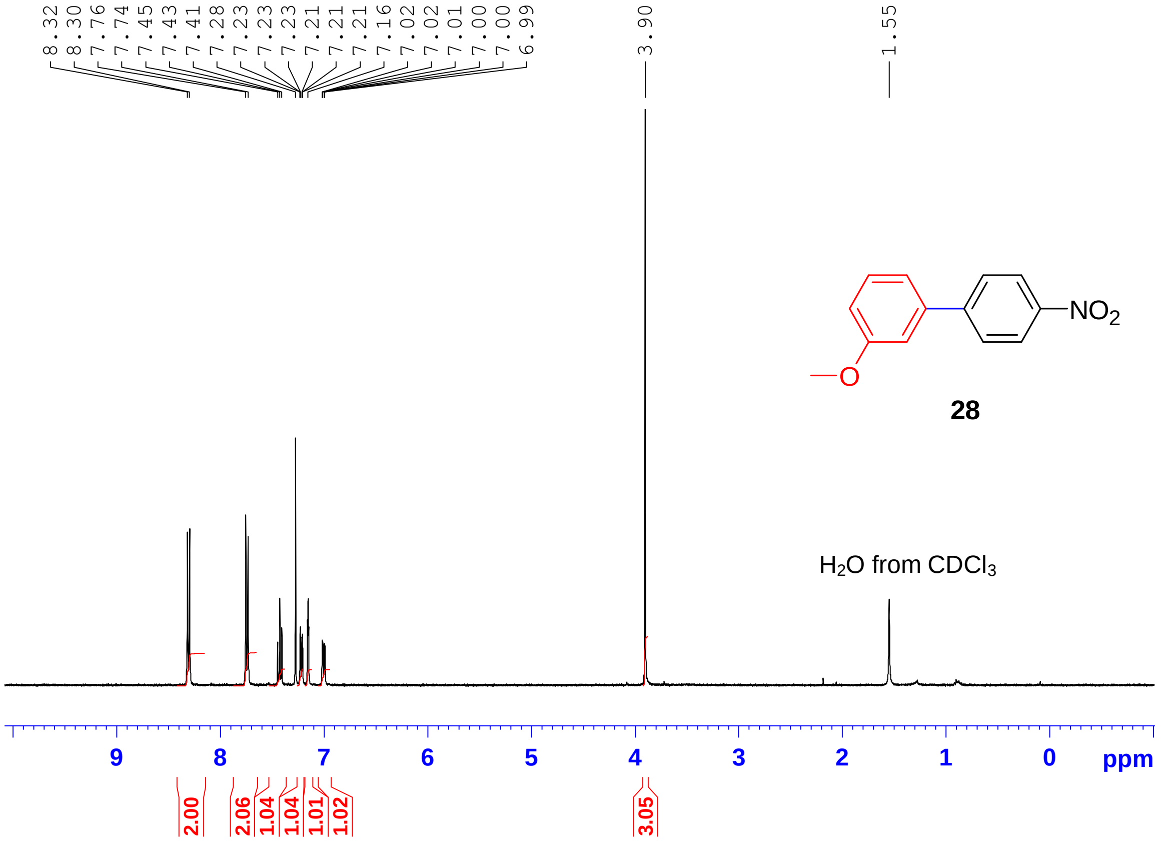
8.32 8.30 7.76 7.74 7.45 7.43 7.41 7.28 7.23 7.23 7.23 7.21 7.21 7.21 7.16 7.02 7.02 7.01 7.00 7.00 6.99
3.90
1.55
NO2
O
28
H2O from CDCl3
9 8 7 6 5 4 3 2 1 0 ppm
2.00 2.06 1.04 1.04 1.01 1.02
3.05

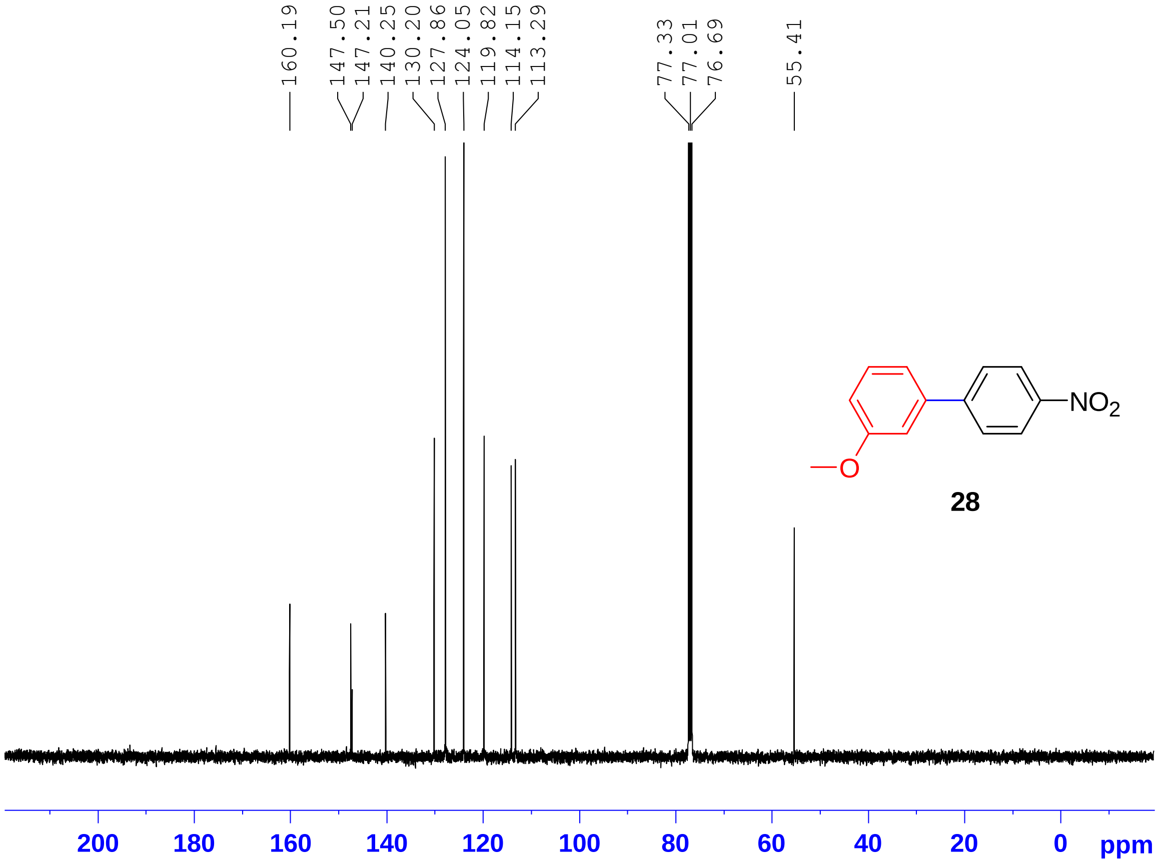
160.19
147.50 147.21 140.25 130.20 127.86 124.05 119.82 114.15 113.29
77.33 77.01 76.69
55.41
NO2
O
28
200 180 160 140 120 100 80 60 40 20 0 ppm

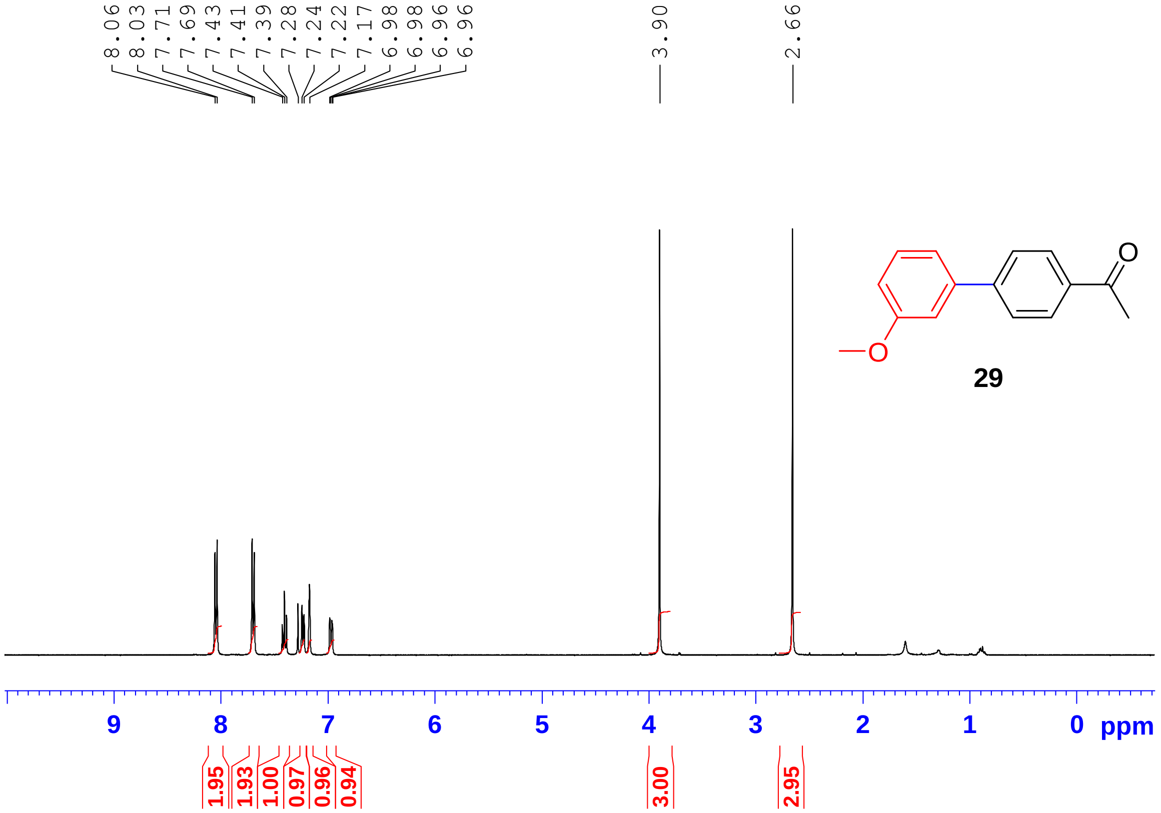
8.06 8.03 7.71 7.69 7.43 7.41 7.39 7.28 7.24 7.22 7.17 6.98 6.98 6.96 6.96
3.90
2.66
O
29
9 8 7 6 5 4 3 2 1 0 ppm
1.95 1.93 1.00 0.97 0.96 0.94
3.00
2.95

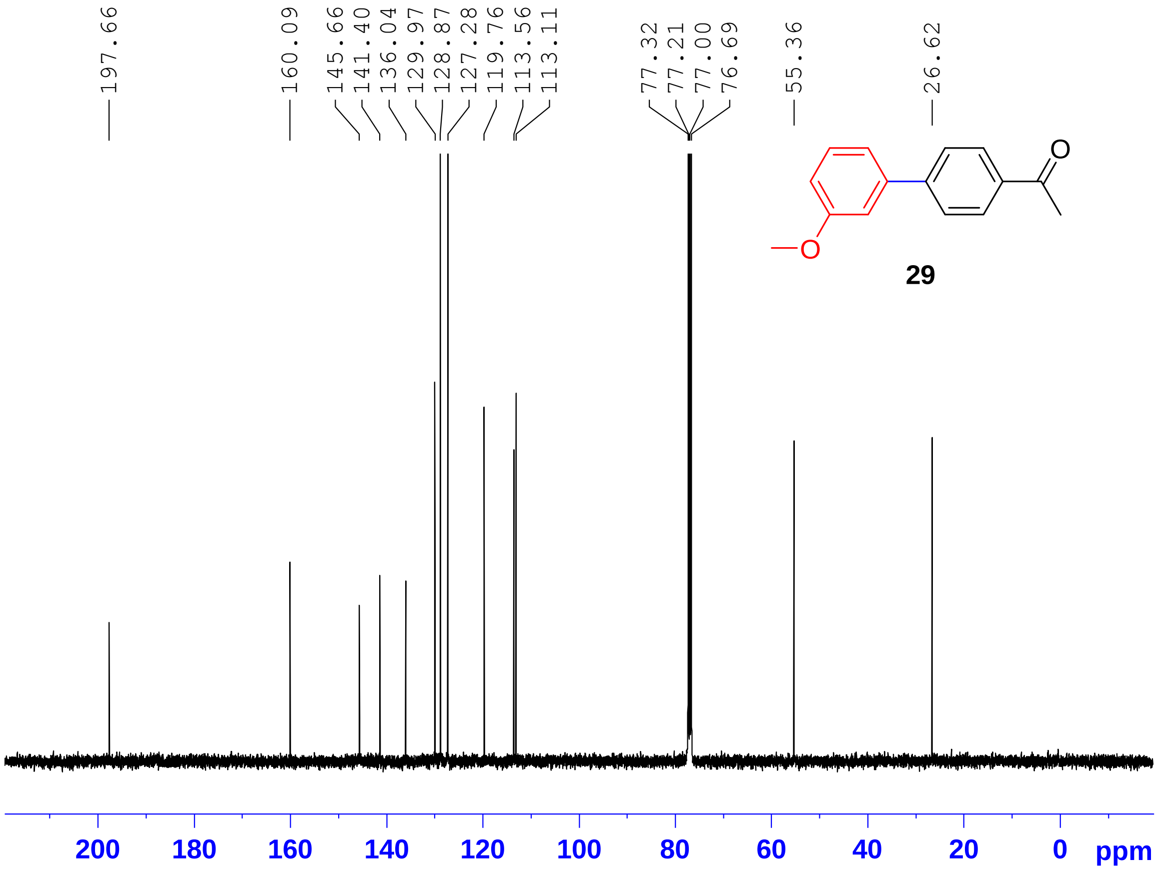
197.66
160.09
145.66 141.40 136.04 129.97 128.87 127.28 119.76 113.56 113.11
77.32 77.21 77.00 76.69
55.36
26.62
O
29
200 180 160 140 120 100 80 60 40 20 0 ppm

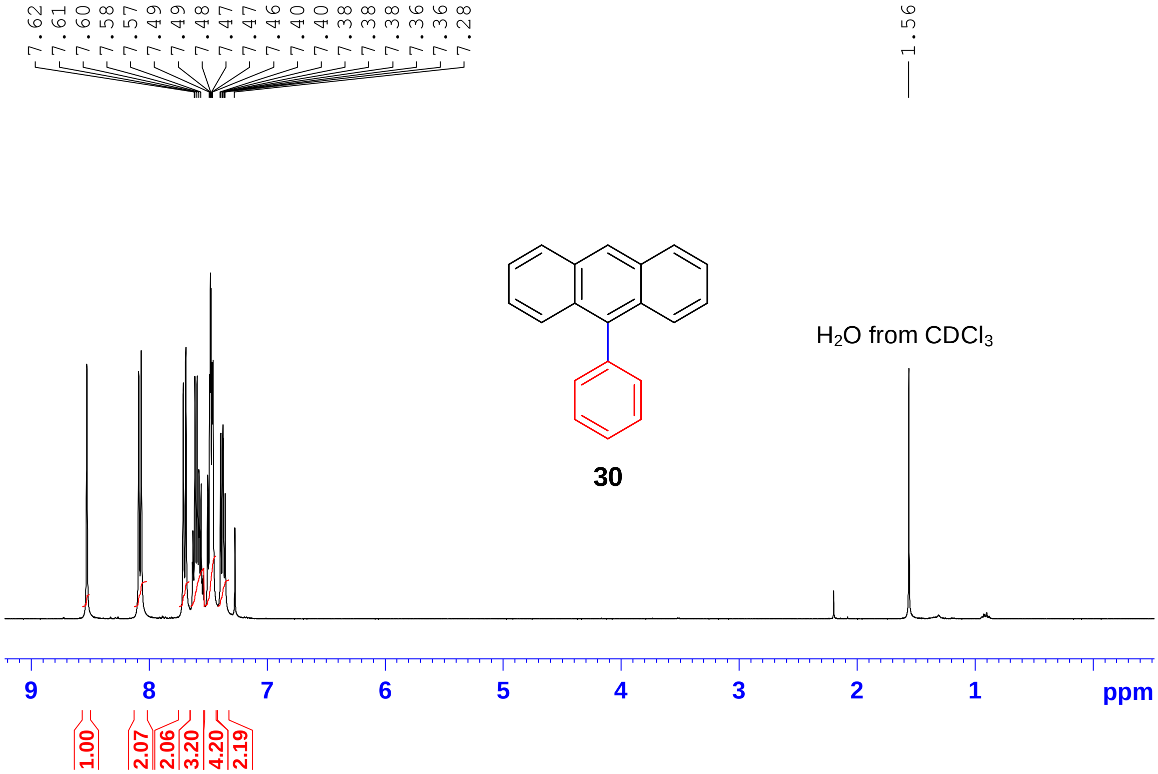
7.62
7.61
7.60
7.58
7.57
7.49
7.49
7.48
7.47
7.47
7.46
7.40
7.40
7.38
7.38
7.38
7.36
7.36
7.28
1.56
H_2O from $CDCl_3$
30
9 8 7 6 5 4 3 2 1 ppm
1.00
2.07
2.06
3.20
4.20
2.19

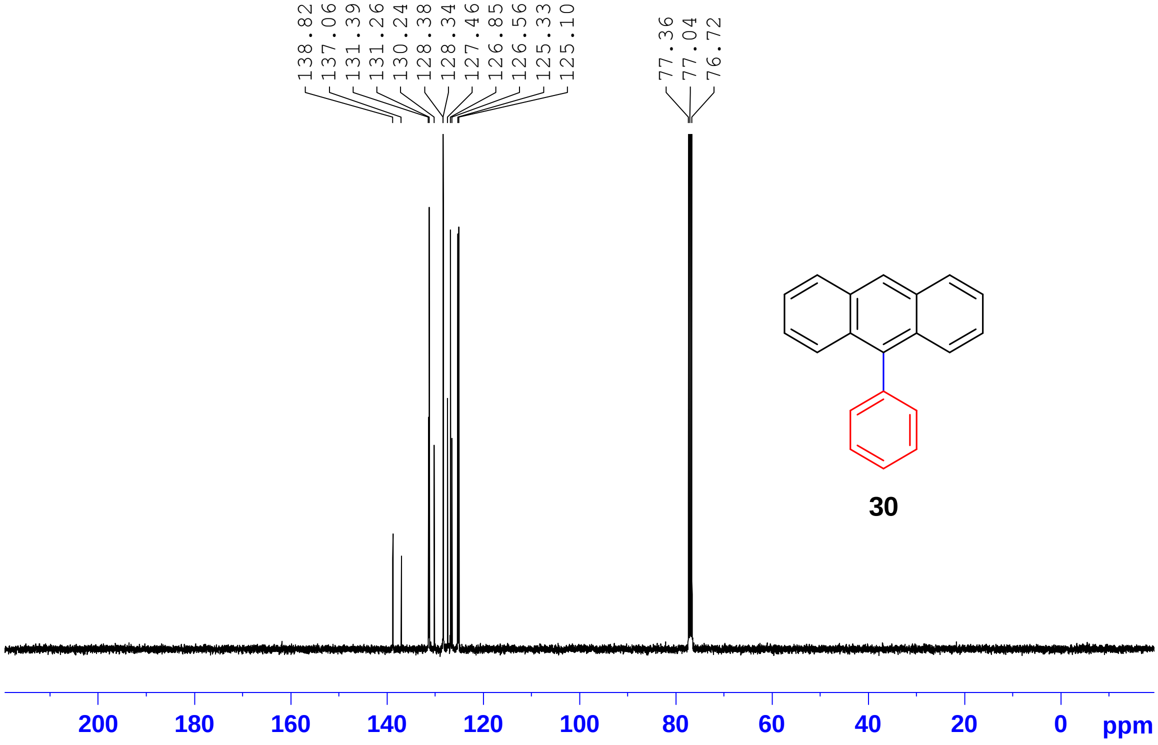
138.82
137.06
131.39
131.26
130.24
128.38
128.34
127.46
126.85
126.56
125.33
125.10
77.36
77.04
76.72
30
200 180 160 140 120 100 80 60 40 20 0 ppm

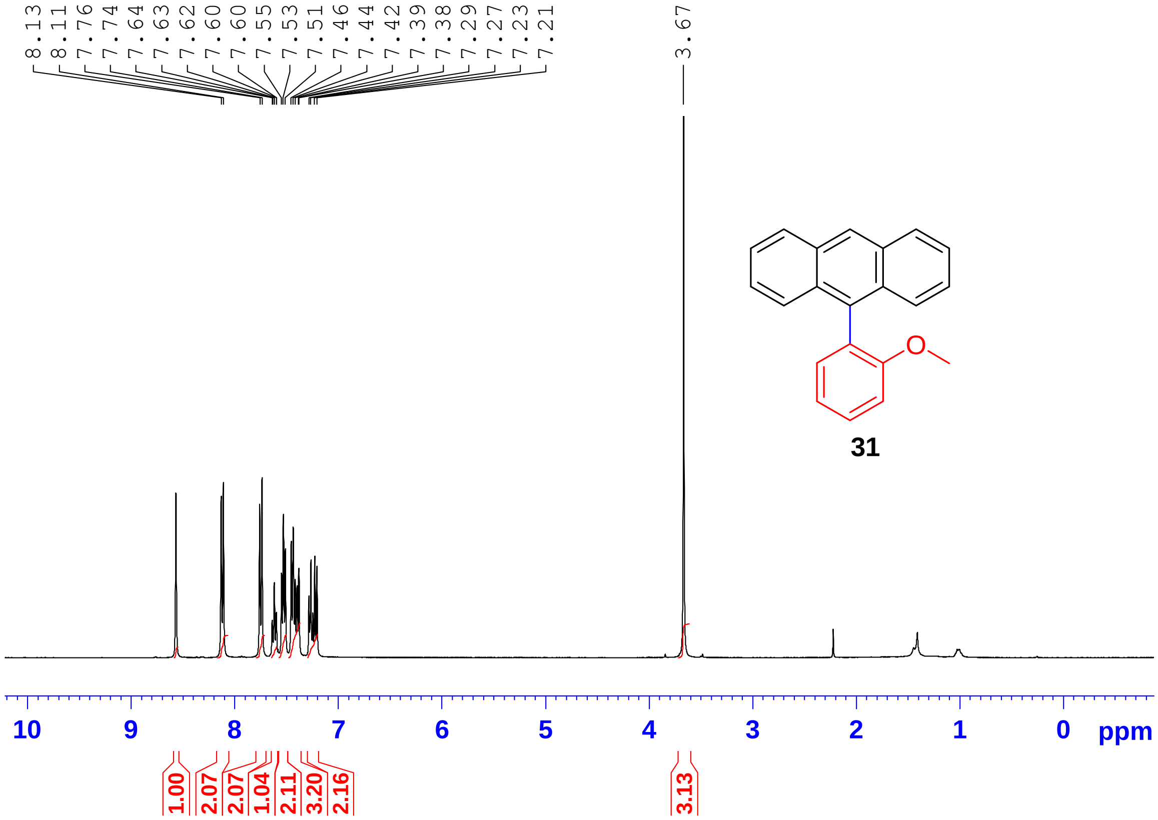
8.13
8.11
7.76
7.74
7.64
7.63
7.62
7.60
7.60
7.55
7.53
7.51
7.46
7.44
7.42
7.39
7.38
7.29
7.27
7.23
7.21
3.67
31
10
9
8
7
6
5
4
3
2
1
0
ppm
1.00
2.07
2.07
1.04
2.11
3.20
2.16
3.13

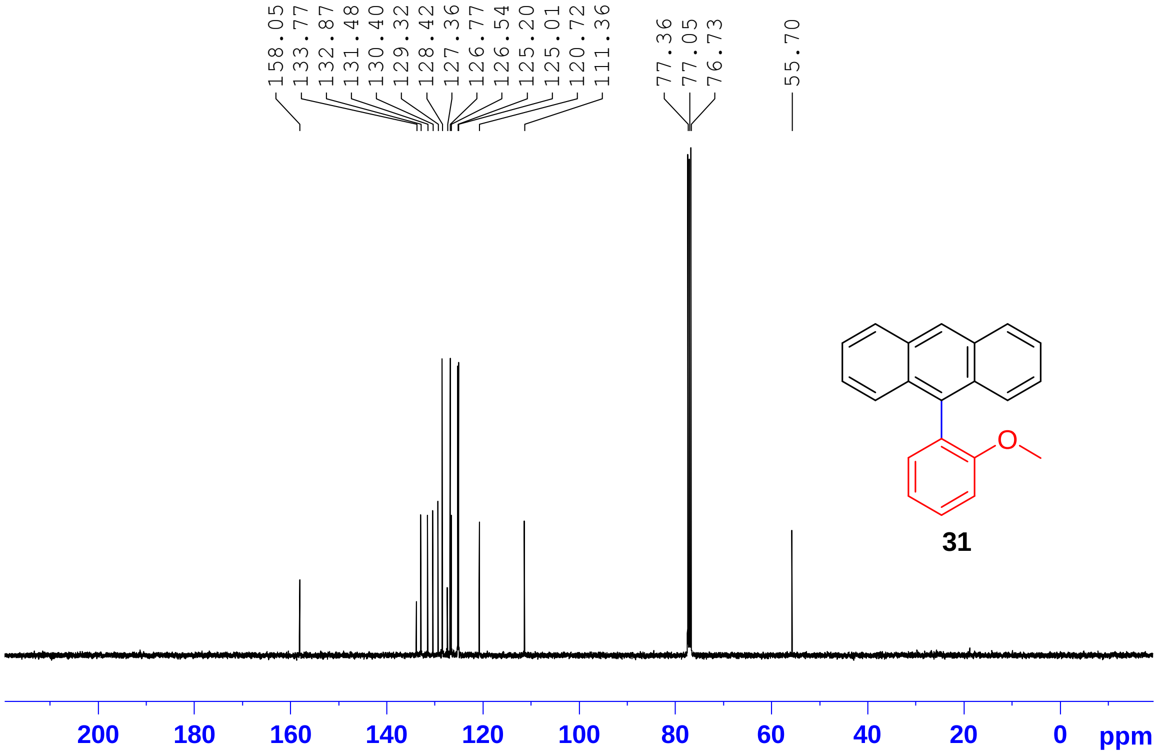
158.05
133.77
132.87
131.48
130.40
129.32
128.42
127.36
126.77
126.54
125.20
125.01
120.72
111.36
77.36
77.05
76.73
55.70
31
200
180
160
140
120
100
80
60
40
20
0
ppm

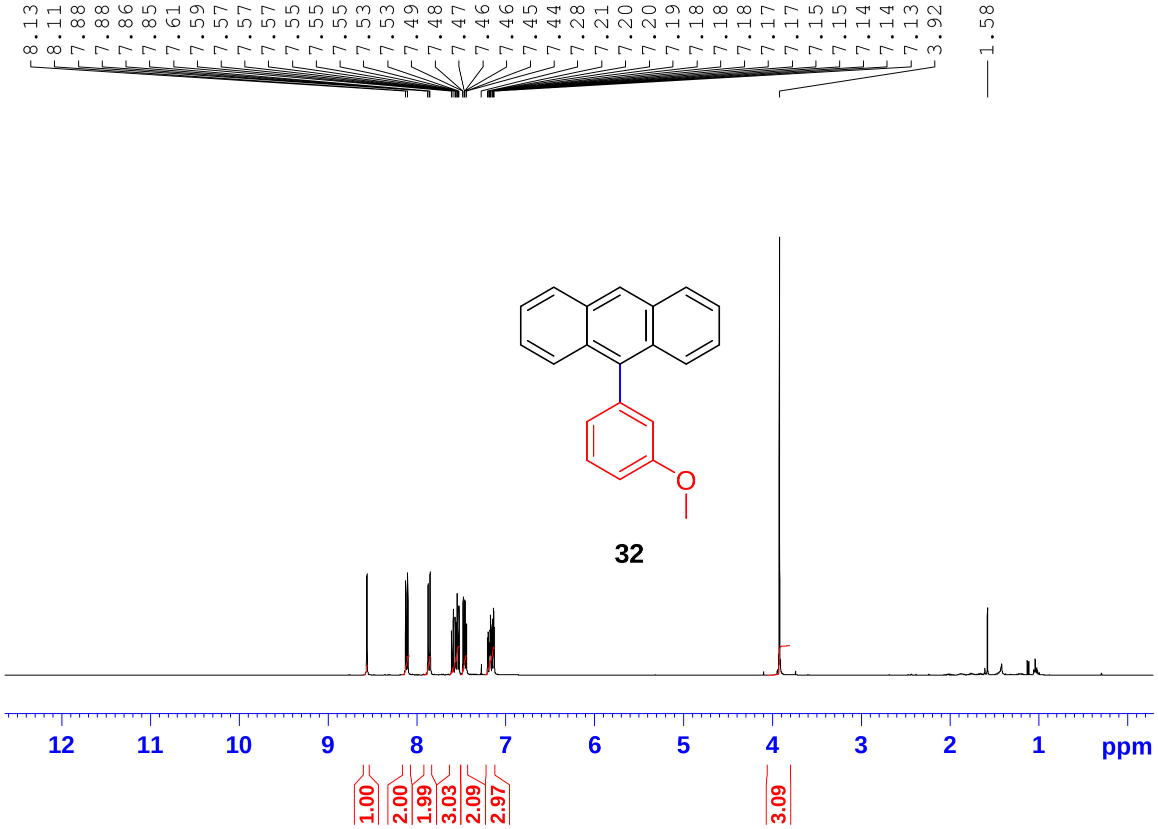
8.13 8.11 7.88 7.88 7.86 7.85 7.61 7.59 7.57 7.57 7.57 7.55 7.55 7.55 7.53 7.53 7.49 7.48 7.47 7.46 7.46 7.45 7.44 7.28 7.21 7.20 7.20 7.19 7.18 7.18 7.18 7.17 7.17 7.15 7.15 7.14 7.14 7.13 3.92 1.58
32
12 11 10 9 8 7 6 5 4 3 2 1 ppm
1.00 2.00 1.99 3.03 2.09 2.97 3.09

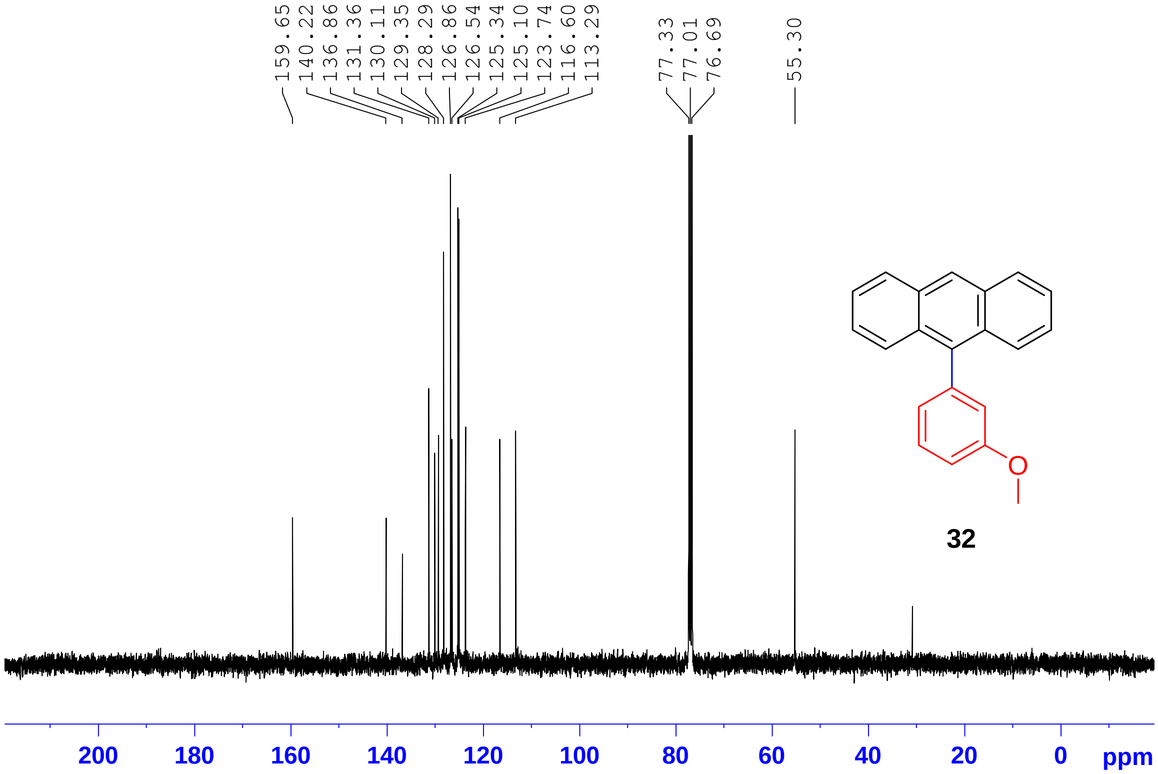
159.65 140.22 136.86 131.36 130.11 129.35 128.29 126.86 126.54 125.34 125.10 123.74 116.60 113.29
77.33 77.01 76.69
55.30
32
200 180 160 140 120 100 80 60 40 20 0 ppm
